# A novel botybirnavirus with a unique satellite dsRNA causes latent infection in *Didymella theifolia* isolated from tea plants

**DOI:** 10.1128/spectrum.00033-23

**Published:** 2023-11-14

**Authors:** Liangchao Ye, Xinyu Shi, Yunqiang He, Jiao Chen, Qingeng Xu, Karim Shafik, Lanning Fu, Yumeng Yin, Ioly Kotta-Loizou, Wenxing Xu

**Affiliations:** 1 National Key Laboratory for Germplasm Innovation & Utilization of Horticultural Crops, Wuhan, China; 2 Hubei Hongshan Laboratory, Wuhan, China; 3 College of Plant Science and Technology, Huazhong Agricultural University, Wuhan, China; 4 Key Lab of Plant Pathology of Hubei Province, Wuhan, China; 5 Department of Plant Pathology, Faculty of Agriculture, Alexandria University, Alexandria, Egypt; 6 Department of Life Sciences, Faculty of Natural Sciences, Imperial College London, London, United Kingdom; 7 Department of Clinical, Pharmaceutical and Biological Science, School of Life and Medical Sciences, University of Hertfordshire, Hatfield, United Kingdom; Emory University School of Medicine, Atlanta, Georgia, USA

**Keywords:** mycovirus, botybirnavirus, Didymella theifolia botybirnavirus 1, satellite dsRNA, *Didymella theifolia*, *Camellia sinensis*

## Abstract

The unique, recently discovered fungus *Didymella theifolia* specifically infects local varieties of tea plant *Camellia sinensis* in China, and therefore, the characterization of its mycoviruses is important. Three double-stranded (ds) RNAs (1, 2, and 3, with 6,338, 5,910, and 727 bp in size, respectively) were identified in the avirulent *D. theifolia* strain CJP4-1, which exhibits normal growth and morphology. Characterization of these double-stranded RNAs (dsRNAs) revealed that the two largest elements are the genomic components of a novel botybirnavirus, tentatively named Didymella theifolia botybirnavirus 1 (DtBRV1). Conversely, dsRNA3 shares no detectable similarity with sequences deposited in public databases but has high similarity with the 5′-terminal regions of dsRNAs 1 and 2 and contains a duplicated region encoding a putative small peptide. All three dsRNAs are encapsidated in isometric virions *ca*. 40 nm in diameter, supporting the notion that dsRNA3 is a DtBRV1 satellite. SDS-polyacrylamide gel electrophoresis in combination with peptide mass fingerprint analysis revealed that the DtBRV1 capsid protein consists of polypeptides encoded by the 5′-terminal regions of both genomic components dsRNA1 and dsRNA2. Vertical transmission of DtBRV1 through conidia is efficient, while its horizontal transmission from CJP4-1 to other strains was not detected. DtBRV1, with or without dsRNA3, has no obvious effects on fungal growth and virulence, as illustrated following transfection of the virulent *D. theifolia* strain JYC1-6. In summary, DtBRV1 exhibits unique molecular traits and contributes to our understanding of mycovirus diversity.

**IMPORTANCE:**

A novel botybirnavirus, infecting the tea plant pathogen *Didymella theifolia* and tentatively named Didymella theifolia botybirnavirus 1 (DtBRV1), together with an additional double-stranded RNA (dsRNA), was characterized. DtBRV1 comprises two dsRNAs (1 and 2) encapsidated in isometric virions, while dsRNA3 is a satellite. The satellite represents a unique specimen since it contains a duplicated region and has high similarity to the two botybirnavirus dsRNAs, supporting the notion that it most likely originated from a deficient genomic component. The biological characteristics of DtBRV1 were further determined. With their unique molecular traits, DtBRV1 and its related dsRNA expand our understanding of virus diversity, taxonomy, and evolution.

## INTRODUCTION

Mycoviruses (fungal viruses) have been widely reported in all major taxa of fungi, including filamentous fungi, yeasts, and oomycetes (1 – 5), since they were first discovered in the mushroom *Agaricus bisporus* in 1962 (6). Based on the nature and function of their genomic nucleic acids, mycoviruses have been taxonomically grouped into 22 families by the International Committee on Taxonomy of Viruses (ICTV, https://talk.ictvonline.org), with some mycoviruses still remaining unclassified (7, 8). Established families accommodating mycoviruses include 12 with positive-sense single-stranded RNA (+ssRNA) genomes (*Alphaflexiviridae*, *Barnaviridae*, *Botourmiaviridae*, *Deltaflexiviridae*, *Endornaviridae*, *Fusariviridae*, *Gammaflexiviridae*, *Hadakaviridae*, *Hypoviridae*, *Mitoviridae*, *Narnaviridae*, and *Yadokariviridae*) (9), 8 with double-stranded RNA (dsRNA) genomes (*Alternaviridae*, *Chrysoviridae*, *Megabirnaviridae*, *Partitiviridae*, *Polymycoviridae*, *Quadriviridae*, *Spinoreoviridae*, and *Totiviridae*) (10), 1 with negative-sense ssRNA (–ssRNA) genomes (*Mymonavirida*e) (7), and 1 with ssDNA genomes (*Genomoviridae*) (11). Many of these mycoviruses are parasitic to their host fungi in a latent manner, while some can dramatically attenuate the virulence of host fungi or even change their lifestyle from a pathogen into an endophyte and may be used as biocontrol agents against fungal diseases (11 – 14), exemplified by successful control of chestnut blight by Cryphonectria hypovirus 1 (CHV1) in Europe (6).

The numbers of characterized mycoviral genomes have largely increased in recent years with the aid of next-generation sequencing (NGS), following the development of this method in the second half of the 2000s. However, knowledge on mycoviruses associated with fungi infecting tea plants [*Camellia sinensis* (L.) O. Kuntze] is still very limited. Tea is an important crop in tropical and subtropical regions, has gained favor with consumers as a healthy beverage, and is ranked as the most consumed beverage worldwide after water during the past decades (15, 16). Importantly, tea plants originated in China and have been cultivated for over 3,000 years, representing an ancient species in Asia. The characterization of their mycovirome would open a window to a unique microbiological community, as exemplified by the first filamentous dsRNA virus (17) and the first dsRNA mycovirus that changes fungal lifestyle from a pathogen into an endophyte (13). *Didymella theifolia* W.X. Xu and Y.Q. He is a newly identified fungus that infects tea plants causing tea leaf brown-black spot disease, locally known in Chinese as “chixingbing.” This disease is characterized by tiny blackish- or reddish-brown spots on tender leaves and affects tea quality leading to a bitter and astringent flavo (18). To date, no mycoviruses have been identified in this fungal genus.

*Botybirnavirus* is a mycoviral genus whose first member was identified in 2012; it has not been assigned to any families by the ICTV although *Botybirnaviridae* was originally proposed (19). Up to now, only six members belonging to this genus have been identified and characterized in three phytopathogenic fungi, including *Botrytis porri*, *Sclerotinia sclerotiorum*, and *Alternaria* spp., and all have bipartite genomes comprising two dsRNA components with similar sizes encapsidated in isometric virions (19 – 23). An RNA-dependent RNA polymerase (RdRp) domain is encoded by one of the dsRNAs, while the provenance of the capsid protein (CP) remains undetermined. Overall, this mycoviral genus remains understudied.

Here, a bipartite dsRNA mycovirus with a unique satellite was detected in *D. theifolia* and identified as a novel botybirnavirus based on sequence and phylogenetic analysis. This botybirnavirus, together with its satellite, presents unusual molecular and biological traits and contributes useful information for a better understanding of the mycovirome.

## RESULTS

### *D. theifolia* strain CJP4-1 harbors three dsRNAs

**Fig 1:**
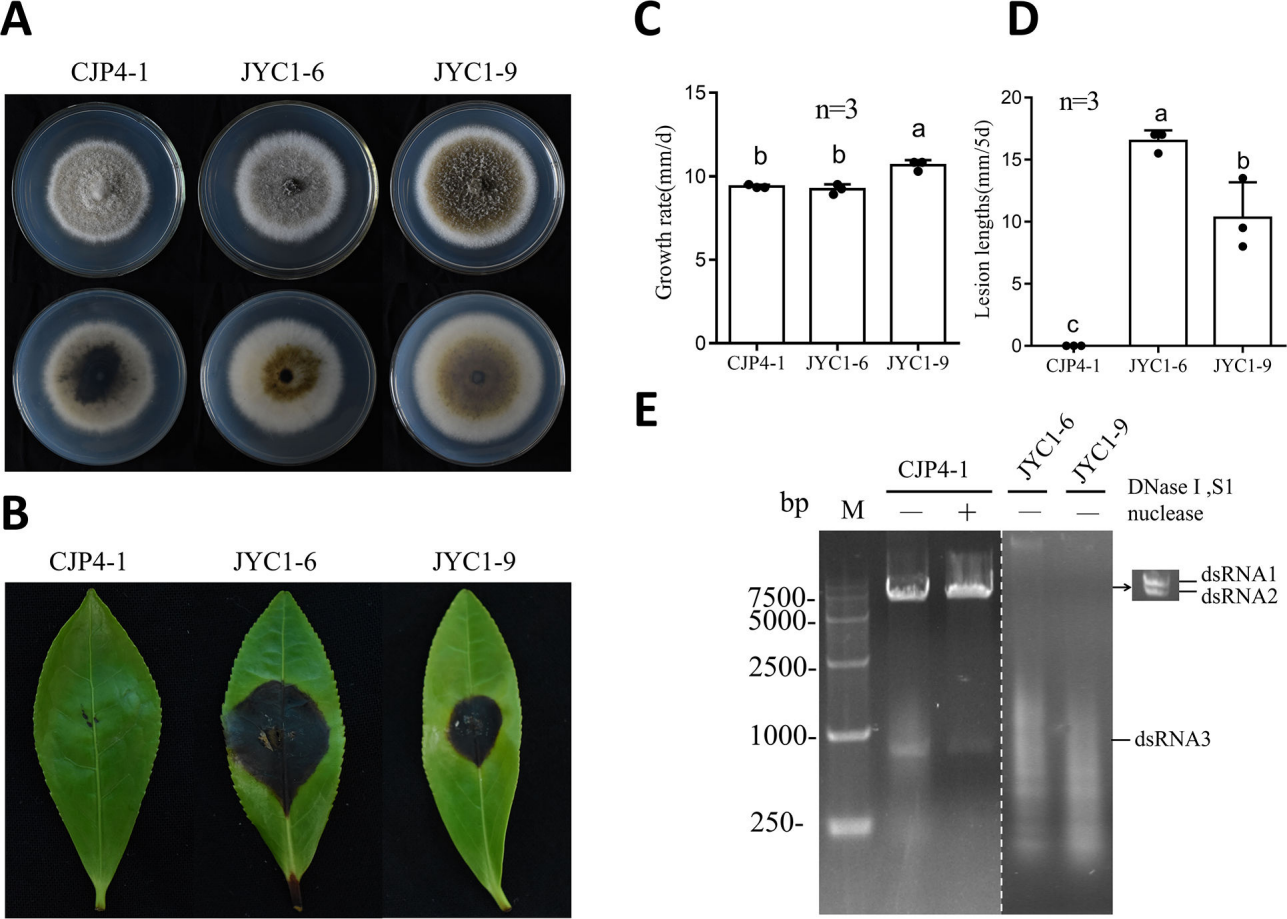
Morphology, growth rate, virulence, and extraction electrophoretic profile of *Didymella theifolia* strains CJP4-1, JYC1-6, and JYC1-9. (**A**) Colony morphology of *D. theifolia* strains cultured on potato dextrose agar (PDA) at 25°C for 7 days. (**B**) Virulence of *D. theifolia* strains on detached tea leaves (*Camellia sinensis* var. E’cha no.1) at 5 days post-inoculation (dpi) following wounding. (**C**) Growth rates *in vitro* and (**D**) lesion lengths *in planta* of *D. theifolia* strains. (**E**) Agarose gel electrophoresis of nucleic acids extracted from *D. theifolia* strains, without (–) or with (+) enzyme treatment by DNase I and S1 nuclease.

**Fig 2:**
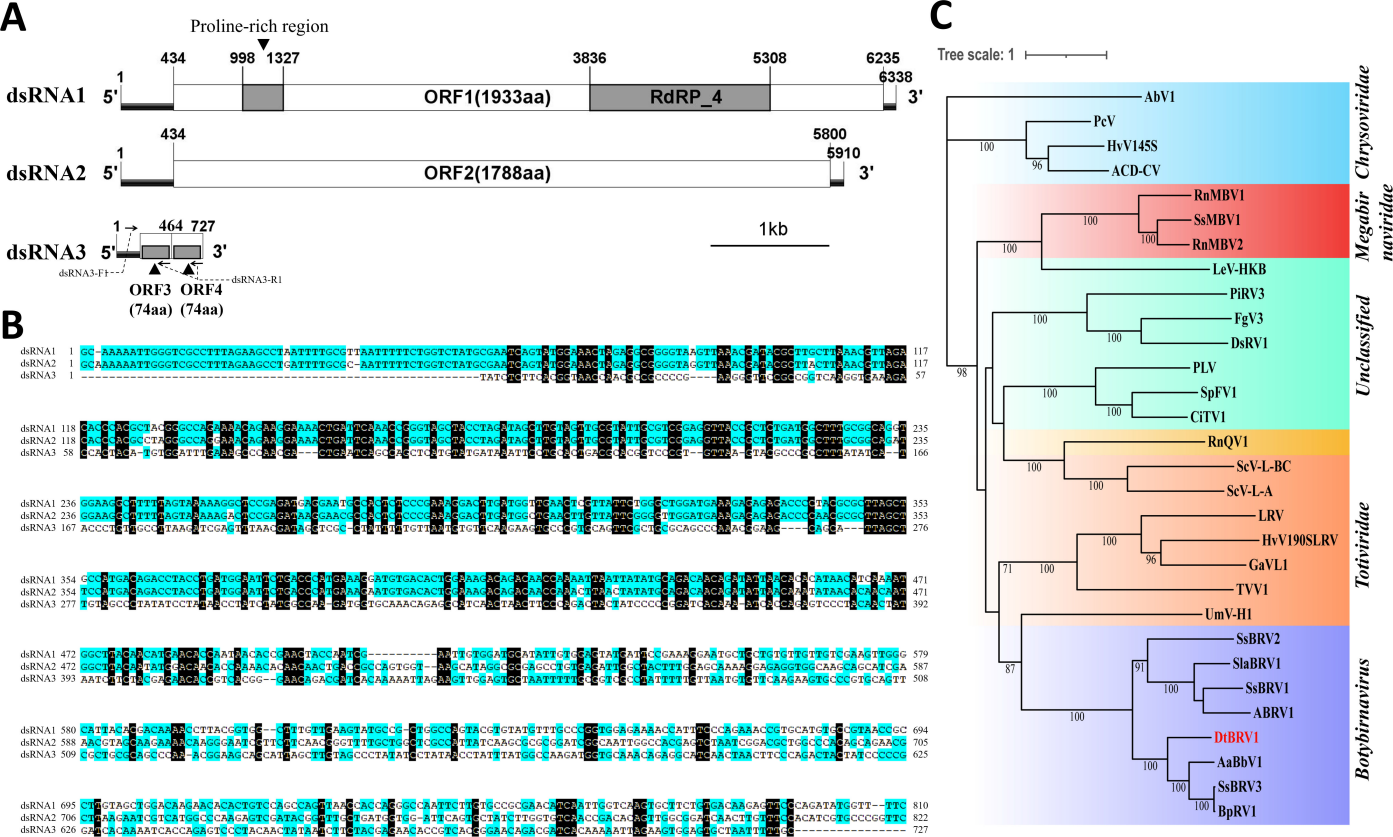
Genomic organization and phylogenetic analysis of Didymella theifolia botybirnavirus 1 (DtBRV1). (**A**) A schematic diagram of the genomic organization of dsRNAs 1 to 3. The start and end positions of untranslated regions (UTRs), open reading frames (ORFs), and an RNA-dependent RNA polymerase-4 superfamily domain are labeled. The two regions of dsRNA1 and dsRNA2 encoding the structural proteins are homologous, as reported for other botybirnaviruses. (**B**) The full-length sequence alignment of dsRNA3 with dsRNAs 1 and 2. Identical nucleotides are highlighted with a dark-blue color. (**C**) A maximum-likelihood phylogenetic tree constructed using IQ-TREE based on RdRp sequences of DtBRV1 and related dsRNA viruses. The bootstrap values were deduced based on 1,000 replicates, and those lower than 70% are not shown in the figure. The scale bar indicates a genetic distance of 1. Names, abbreviations, and other related information of the viruses are listed in Table 1.

*D. theifolia* strain CJP4-1 showed similar colony morphology to *D. theifolia* strains JYC1-6 and JYC1-9 when grown *in vitro*, production of darker pigments notwithstanding (Fig. 1A and C). However, CJP4-1 exhibited no virulence *in vivo* when using a detached tea leaf pathogenicity assay, since no lesions were observed on tea leaves inoculated with CJP4-1, in contrast to the large lesions ranging from 8.0 to 17.0 mm in length observed with JYC1-6 and JYC1-9 (Fig. 1B and D). To investigate whether a mycovirus was responsible for the attenuated virulence of CJP4-1, the mycelia of these three strains were subjected to dsRNA extraction followed by agarose gel electrophoresis. Three dsRNAs, resistant to digestion with DNase I and S1 nuclease and termed dsRNAs 1, 2, and 3 according to their decreasing sizes, were observed in CJP4-1; however, no dsRNA bands were detected in JYC1-6 and JYC1-9 (Fig. 1E). The sequences of the full-length cDNAs derived from dsRNAs 1 and 2 were determined by next-generation sequencing in combination with RNA ligase-mediated rapid amplification of cDNA ends (RLM-RACE) protocols. The sequence of dsRNA3 was obtained by ligation with the PC3-T7loop adaptor followed by reverse transcription and amplification with the complementary PC2 primer (Fig. 2A).

### dsRNAs 1 and 2 comprise the genome of a novel botybirnavirus

dsRNAs 1 and 2 are 6,338 and 5,910 bp, respectively, in length, each predicted to encode an open reading frame flanked by untranslated regions. The 5′-UTRs of both dsRNAs are 433 bp in length and share a high sequence similarity of 94% (Fig. 2B; Fig. S1A). The 3′-UTRs of dsRNAs 1 and 2 are 103 and 110 bp, respectively, in length, and their sequence similarity is 54% (Fig. S1B).

The predicted ORFs of dsRNAs 1 and 2 were termed ORF1 (positioned at 434–6,235 bp) and ORF2 (positioned at 434–5,800 bp), respectively. ORF1 encodes a protein 1,933 aa in length with an estimated molecular mass of 217 kDa, containing a proline-rich domain at position 998–1,326 bp and an RNA-dependent RNA polymerase-4 superfamily structural domain at position 3,836–5,308 bp. The predicted RdRp sequence harbors eight conserved motifs (I–VIII), similar to those of other dsRNA viruses (Fig. S2). BLASTp searches of public databases revealed that the ORF1-encoded protein had the highest similarity to proteins encoded by Sclerotinia sclerotiorum botybirnavirus 3 (SsBRV3, accession no. AWY10943.1, *E*-value 0.0, coverage 98%, identity 47%). A maximum-likelihood phylogenetic tree constructed using related RdRps (Table 1) revealed that the ORF1-encoded protein clusters together with members of genus *Botybirnavirus* and is most closely related to SsBRV3, Alternaria alternata botybirnavirus 1 (AaBRV1), and Botrytis porri botybirnavirus 1 (BpRV1) (Fig. 2D). ORF2 encodes a putative protein 1,788 aa in length with an estimated molecular mass of 198 kDa and shows high similarity to proteins encoded by SsBRV3, BpBRV1, and AaBRV1 (*E*-value 0.0, coverage 95%–99%, identity 40%). Taken together, these results suggest that dsRNAs 1 and 2 are the genomic components of a novel botybirnavirus, tentatively named Didymella theifolia botybirnavirus 1.

**TABLE 1:** The information for the viruses used for phylogenetic analysis

Genus or family	Species	Abbreviation	RdRp (aa)	Accession no.
*Botybirnavirus*	Alternaria botybirnavirus 1^*a*^	ABRV1	1723	ARQ84132.1
	Alternaria alternata botybirnavirus 1^*a*^	AaBbV1	1878	BBH54877.1
	Botrytis porri RNA virus 1^*a*^	BpRV1	1902	YP_006390636.1
	Didymella theifolia botybirnavirus 1^*a*^	DtBRV1	1933	OQ078673.1
	Sclerotinia sclerotiorum botybirnavirus 1^*a*^	SsBRV1	1925	YP_009141011.1
	Sclerotinia sclerotiorum botybirnavirus 2^*a*^	SsBRV2	1868	AMT92139.1
	Sclerotinia sclerotiorum botybirnavirus 3^*a*^	SsBRV3	1902	UOJ41050.1
	Soybean leaf-associated botybirnavirus 1	SlaBRV1	792	ALM62244.1
*Chrysoviridae*	Agaricus bisporus virus 1	AbV1	1078	CAA64144.1
	Amasya cherry disease associated chrysovirus	ACD-CV	1087	YP_001531163.1
	Helminthosporium victoriae 145S virus	HvV145S	1086	YP_052858.1
	Penicillium chrysogenum virus	PcV	1117	YP_392482.1
*Megabirnaviridae*	Rosellinia necatrix megabirnavirus 1	RnMBV1	1111	YP_003288763.1
	Rosellinia necatrix megabirnavirus 2	RnMBV2	1108	YP_009227124.1
	Sclerotinia sclerotiorum megabirnavirus 1	SsMBV1	1117	YP_009143529.1
*Quadriviridae*	Rosellinia necatrix quadrivirus 1	RnQV1	1310	YP_005097975.1
*Totiviridae*	Gremmeniella abietina RNA virus L1	GaVL1	1601	NP_624332.2
	Helminthosporium victoriae virus 190S	HvV190S	835	NP_619670.2
	Leishmania RNA virus 1–1	LRV	874	NP_041191.1
	Saccharomyces cerevisiae Virus L A	ScV-L-A	731	NP_620495.1
	Saccharomyces cerevisiae virus L-BC (La)	ScV-L-BC	863	NP_042581.1
	Trichomonas vaginalis virus 1	TVV1	756	AAA62868.1
	Ustilago maydis virus H1^*a*^	UmV-H1	1820	NP_620728.1
Unclassified	Circulifer tenellus virus 1	CiTV1	1326	YP_003800003.1
	Diplodia scrobiculata RNA virus 1	DsRV1	1110	YP_003359178.1
	Fusarium graminearum dsRNA mycovirus-3	FgV3	1311	YP_003288789.1
	Lentinula edodes mycovirus HKB	LeV-HKB	1426	BAG71788.2
	Persimmon latent virus	PLV	1277	YP_009025166.1
	Phytophthora infestans RNA virus 3	PiRV3	1011	AEX87902.1
	Spissistilus festinus virus 1	SpFV1	1338	YP_003800001.1

^*a*^ The cap-pol fusion protein or polyprotein of this virus was used for analysis.

dsRNA3 is 727 bp in length and has a duplicated region positioned at 199–463 bp and 464–727 bp (Fig. 2A), containing two identical small ORFs (termed ORFs 3 and 4, respectively) predicted to encode a putative peptide of 74 aa in length (*ca*. 8 kDa); however, production of *bona fide* proteins is unlikely due to the small sizes of the predicted ORFs and a lack of significant similarity of the putative peptides to known proteins, as revealed by BLASTp searches. Similarly, dsRNA3 had no significant similarity with sequences deposited in public databases, as revealed by BLASTn and BLASTx searches. However, sequence alignment of dsRNA3 with dsRNAs 1 or 2 showed that it has a 41% similarity with dsRNA1 at position 57–796 bp and a 37% similarity with dsRNA2 at position 57–806 bp (Fig. 2B).

### dsRNAs 1 to 3 are encapsidated in isometric virions

**Fig 3:**
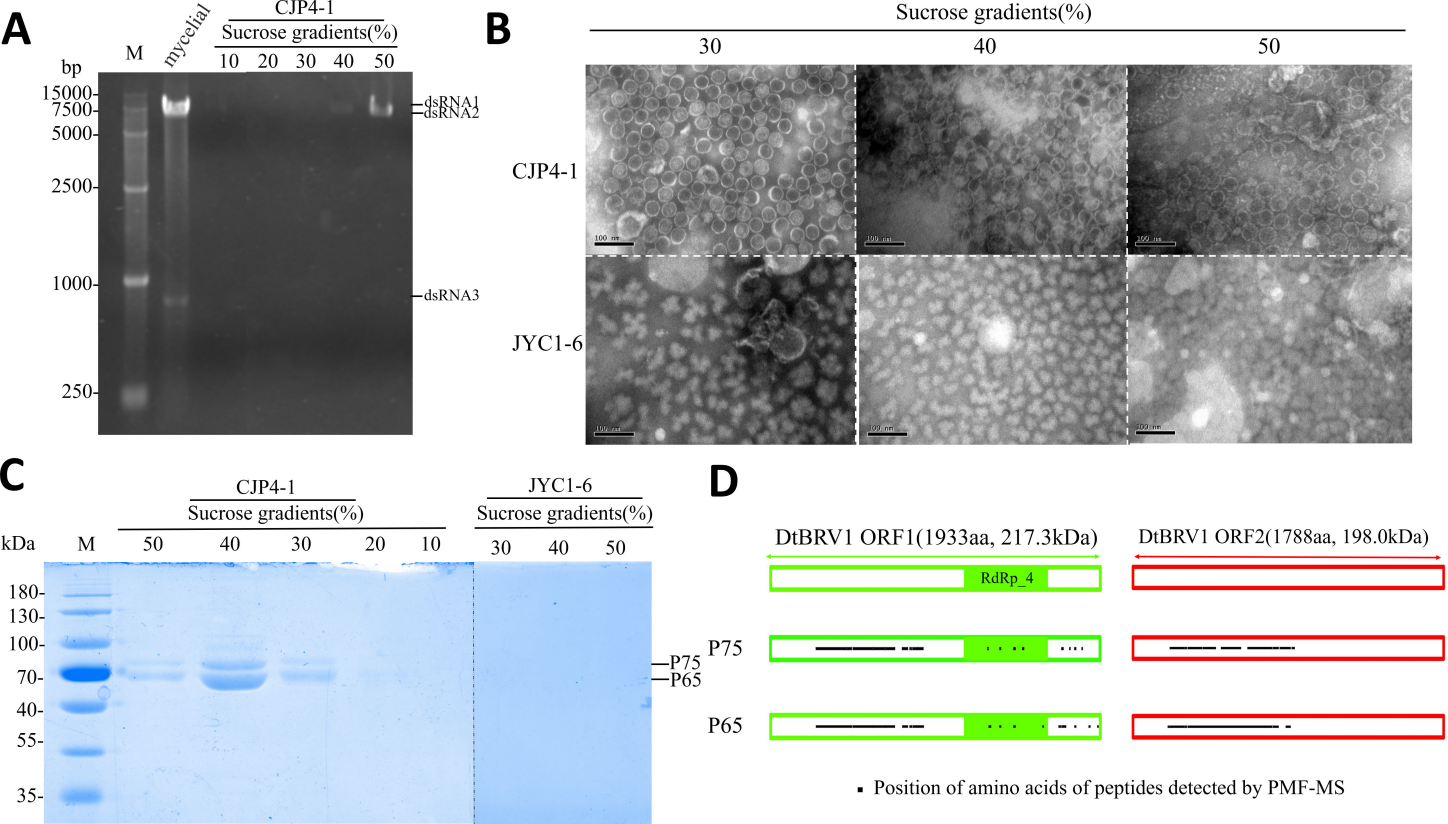
DtBRV dsRNAs, particles, and protein extracted from fractions following sucrose gradient centrifugation. (**A**) Agarose gel electrophoresis of dsRNAs extracted from CJP4-1 mycelia and purified DtBRV1 particles found in 10% to 50% sucrose fractions with 10% increments. M, DNA size marker. (**B**) TEM observation of preparations from DtBRV1-infected strain CJP4-1 and DtBRV1-free strain JYC-1-6 in 30% to 50% sucrose fractions. (**C**) SDS-PAGE of proteins from strains CJP4-1 and JYC1-6 found in 10%–50% sucrose fractions with 10% increments. M, protein %molecular weight marker. (**D**) Peptide mass fingerprinting analysis of proteins in purified VLPs. Predicted coding regions for p75 and p65 are shown. The DtBRV1 ORFs. The green box in ORF1 shows the position of the RdRp-4 domain. Dots in boxes indicate the positions of peptide fragments detected by polypeptide mass fingerprint-mass spectrometry (PMF-MS) (Tables S2 and S3).

Viral particles (VLPs) were purified from CJP4-1 in stepwise sucrose gradients (10%–50%, sucrose increments of 10%). Nucleic acids and proteins from each sucrose gradient fraction were pelleted by ultracentrifugation and subjected to agarose gel electrophoresis and SDS-PAGE, respectively. The results showed that both nucleic acids and proteins were mainly distributed in the 30%–50% sucrose fractions (Fig. 3A through C). Following SDS-PAGE, four protein bands between 60 and 100 kDa were observed, two large faint ones and two small intense ones; these proteins were not detected in JYC1-6 (Fig. 3C). Transmission electron microscopy (TEM) revealed isometric virus-like particles approximately 40 nm in diameter in the 30%–50% sucrose fractions; these VLPs were not observed in JYC1-6 (Fig. 3C).

Since dsRNA3 appeared very faintly following agarose gel electrophoresis (Fig. 3A), its co-precipitation with the other DtBRV1 dsRNAs from sucrose fractions was confirmed by semi-quantitative multiplex reverse transcription-PCR (RT-PCR) analysis following serial dilutions (0-, 30-, and 540-fold), illustrating that dsRNA3 was present together with dsRNA1 and dsRNA2 in sucrose fractions of 30%–50%, predominantly 40% (Fig. S3).

### DtBRV1 structural proteins encoded by both dsRNAs 1 and 2

**Fig 4:**
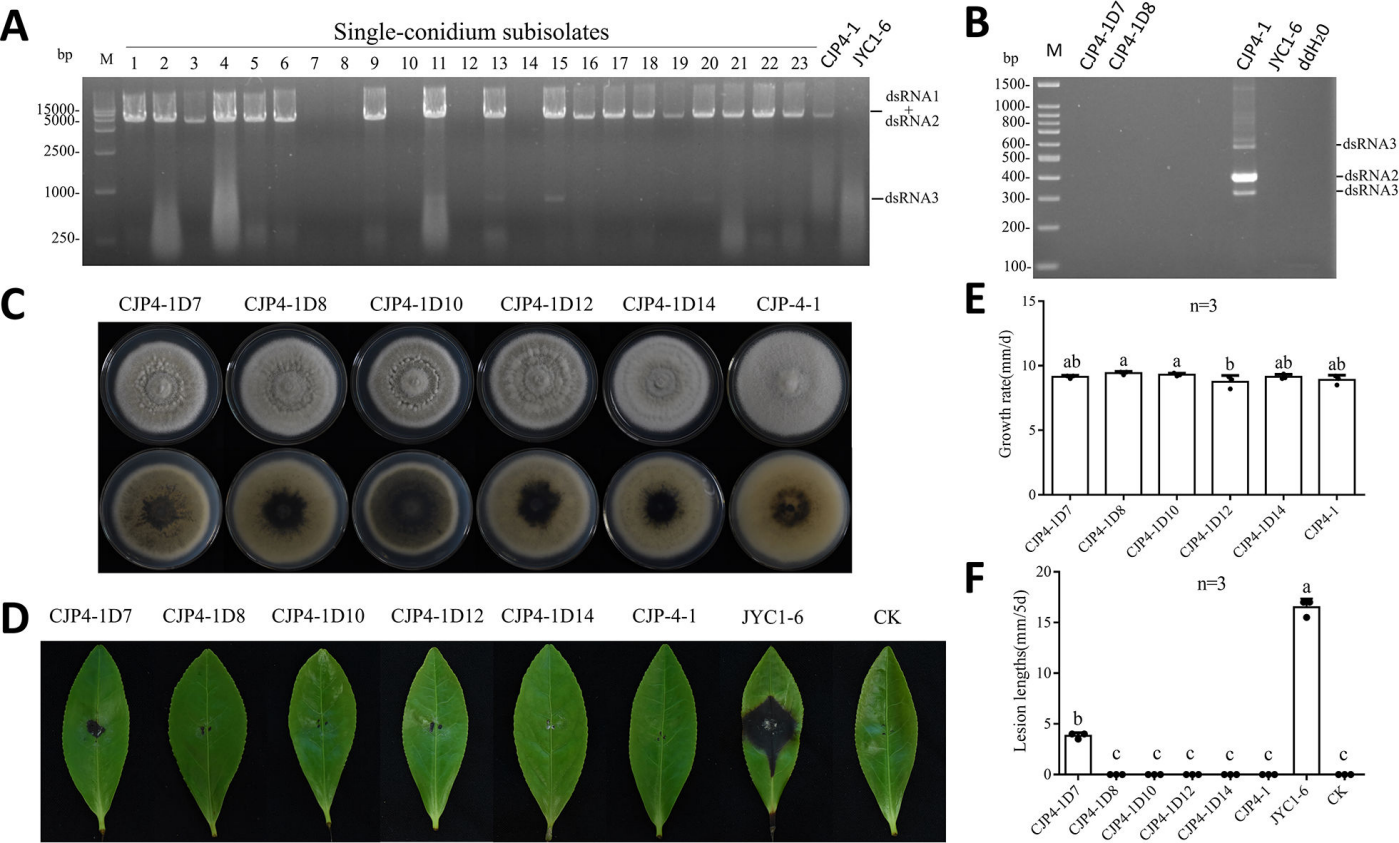
Transmission and effects of DtBRV1 on host morphology, growth, and pathogenicity in CJP4-1. (**A**) Agarose gel electrophoresis of nucleic acids extracted from the mycelia of single-conidium-generated subisolates CJP4-1D1 to CJP4-1D23 (lanes 1 to 23). Strains CJP4-1 and JYC1-6 were used as positive and negative controls, respectively. (**B**) Multiplex RT-PCR identification of the DtBRV1 absence in subisolates CJP4-1D7, -D8, -D10, -D12, and -D14. Strains CJP4-1, JYC1-6, and ddH_2_O were used as a positive, negative, and blank control, respectively. (**C**) Colony morphology of CJP4-1 and its DtBRV1-virus subisolates cultured on PDA at 25°C for 7 days. (**D**) Virulence of CJP4-1 and its DtBRV1-free subisolates on tea leaves (*C. sinensis* var. E’cha no.1). Virulent JYC1-6 was used as positive control and uncolonized PDA plugs as black (CK). (**D**) Growth rate and (**E**) lesion lengths of CJP4-1 and its DtBRV1-free subisolates.

To further verify the identity of the proteins detected by SDS-PAGE analysis, the two intense bands, tentatively termed p75 and p65 based on their approximate sizes, were separately excised from the gel and subjected to peptide mass fingerprinting (PMF) analysis (Fig. 3D; Table S2 and S3). A total of 197 peptide fragments generated from p75 matched the sequence of the ORF1-encoding polypeptide with 32% coverage, dominating at aa positions 267–891, while 271 matched the sequence of the ORF2-encoding polypeptide with 34% coverage, dominating at aa positions 214–931. Similarly, 318 peptide fragments generated from p65 matched the sequence of the ORF1-encoding polypeptide with 34% coverage, dominating at aa positions 267–897, while 271 matched the sequence of the ORF2-encoding polypeptide with 36% coverage, dominating at aa positions 202–907 (Fig. 4B; Tables S2 and S3). These results suggest that both p75 and p65 are predominantly derived from 5′-terminal regions of both ORF1- and ORF2-encoding polypeptides, which are identified as the structural proteins.

### DtBRV1 is efficiently transmitted vertically but not horizontally

**Fig 5:**
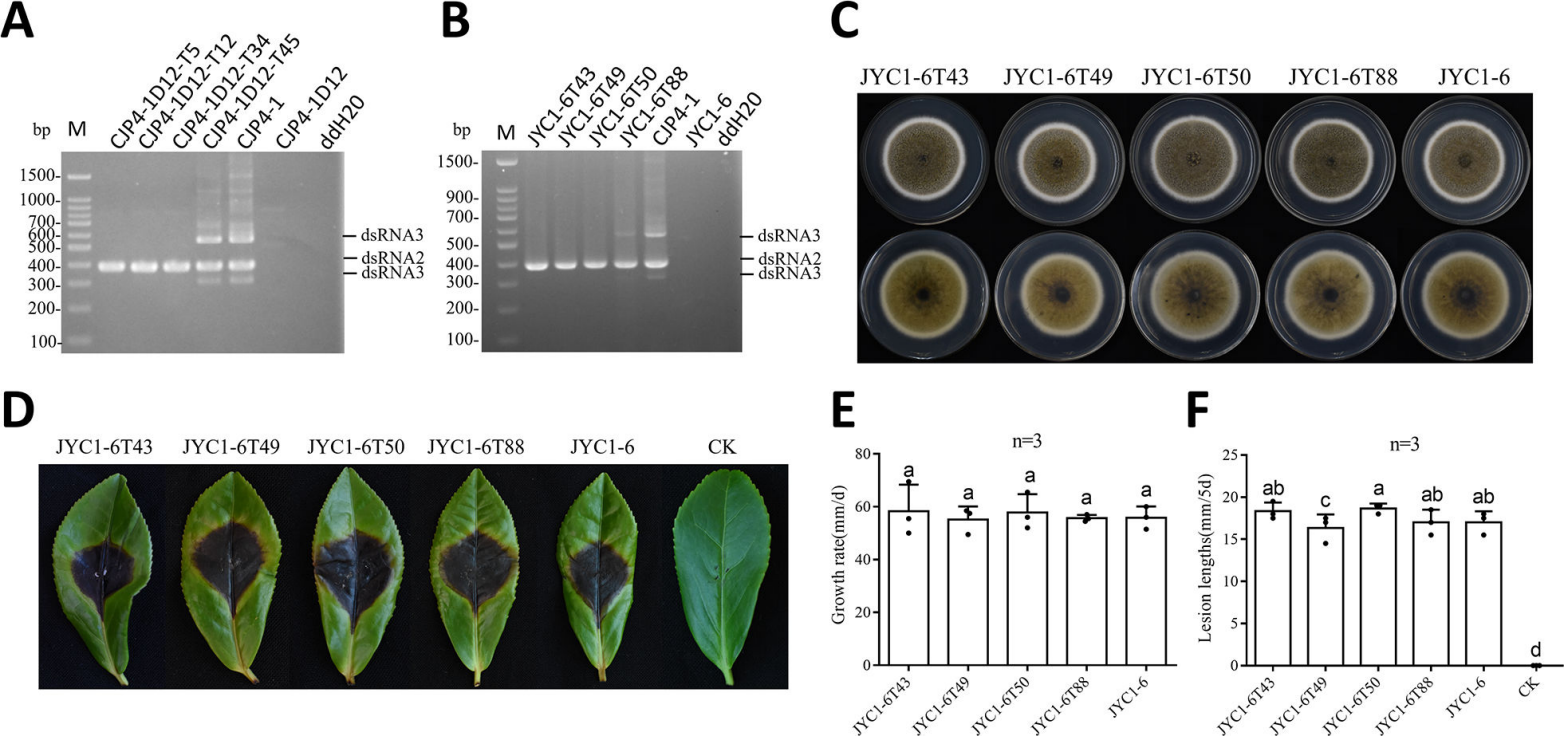
Transmission and effects of DtBRV1 on host morphology, growth, and pathogenicity in JYC1-6. (**A and B**) Multiplex RT-PCR analysis of the co-presence of dsRNAs 2 and 3 in CJP4-1-6D12 and JYC1-6 transfectants using their corresponding primers (Table S1), respectively. The expected PCR product is 390 bp in size for dsRNA2 and both 313 and 578 bp for dsRNA3. (**C**) Colony morphology of DtBRV1-infected transfectants JYC1-6T43, -T49, -T50, -T88, and DtBRV1-free strain JYC1-6 cultured on PDA at 25°C for 8 days. (**D**) Virulence of JYC1-6 and its DtBRV1-infected transfectants on tea leaves (*C. sinensis* var. E’cha no.1). Uncolonized PDA plugs were used as blank (CK). (**E**) Growth rates and (**F**) lesion lengths of JYC1-6 and its DtBRV1-infected transfectants.

To investigate vertical transmission of DtBRV1, CJP4-1 mycelia were inoculated on alfalfa sticks and incubated on PDA plates; the resulting conidia were collected at 15 days post-inoculation, diluted, and cultured on PDA plates for single-conidium colony formation. A total of 23 conidium-generated subisolates (termed CJP4-1D1 to -D23) were selected, transferred on new PDA plates for colony formation, and analyzed for the presence of DtBRV1 dsRNAs by dsRNA extraction. The results showed that 18 out of 23 (78.3%) subisolates carried DtBRV1 dsRNAs 1 and 2, while the remaining five isolates (CJP4-1D7, -D8, -D10, -D12, and -D14) did not (Fig. 4A). Moreover, dsRNA3 was observed together with dsRNAs 1 and 2 in 3 out of 23 (23.1%) subisolates (CJP4-1D13, -D15, and −20D) (Fig. 5A). This was confirmed following RT-PCR detection of dsRNAs 2 and 3 using oligonucleotide primers DtBRV1-F1/-R1 and dsRNA3-F1/-R1, respectively (Fig. 4B; Table S1).

To investigate the horizontal transmission of DtBRV1, the DtBRV1-infected donor strain CJP4-1 and the DtBRV1-free strain JYC1-6 were cultured together for 10 days (Fig. S4A). A total of 15 (3 from each of 5 dual cultures) JYC1-6 mycelium discs far away from the contact area were excised, transferred on new PDA plates for colony formation, and analyzed for the presence of DtBRV1 dsRNAs by dsRNA extraction and RT-PCR. None (0/15) of the subisolates were infected by DtBRV1, potentially due to an incompatibility effect between the two strains (Fig. S4B). Therefore, the DtBRV1-infected donor strain CJP4-1 was cultured together with another DtBRV1-free recipient strain JYC1-9. No incompatibility was observed (Fig. S4A), and 15 JYC1-9 subisolates were analyzed, once more resulting in no subisolates positive for DtBRV1 (Fig. S4B). These results indicated that horizontal transmission of DtBRV1 to other *D. theifolia* strains through hyphal fusion is difficult.

### *D. theifolia* protoplasts are successfully transfected with purified DtBRV1

To complete Koch’s postulates and obtain another, heterologous *D. theifolia* strain carrying DtBRV1, the protoplasts of subisolate CJP4-1D12, previously cured from DtBRV infection, and the DtBRV1-free JYC1-6 strain were transfected with purified DtBRV1 in the presence of PEG 6000. A total of 48 and 107 subisolates were selected from regenerated protoplasts, transferred on new PDA plates for colony formation, and analyzed for the presence of DtBRV1 dsRNAs by dsRNA extraction. The results showed that 19 out of 48 (39.6%) subisolates for CJP4-1D12 and 4 out of 107 (2.8%) subisolates for JYC1-6 (JYC1-6T43, -T49, -T50, and -T88) were infected with DtBRV1 dsRNAs (Fig. S5A and B). This was confirmed following RT-PCR detection of dsRNA2 using oligonucleotide primers DtBRV1-F1/-R1 (Fig. 5A and B; Table S1). Finally, eight DtBRV1-positive transfectants, four derived from CJP4-1D12 and four from JYC1-6, were randomly selected and subjected to multiplex RT-PCR using primer pairs for dsRNA2 and dsRNA3 (Fig. 5A and B; Table S1). The results showed that two subisolates (CJP4-1D12-T45 and JYC1-6T88) were infected by both dsRNA3 and dsRNA2.

### DtBRV1 causes a latent infection

To assess whether DtBRV1 affects the biological traits of its host fungus, DtBRV1-infected parent strain CJP4-1 together with its DtBRV1-free subisolates, including CJP4-1D7, -D8, -D10, -D12, and -D14, was cultured on PDA plates to observe their morphology and measure their growth rate (Fig. 4C and E). All these DtBRV1-free subisolates had similar growth rates but formed more dense aerial mycelia as compared with CJP4-1. Following inoculation on detached tea leaves (*C. sinensis* var. E’cha no.1), all these DtBRV1-free subisolates, similar to CJP4-1, induced no obvious lesions (Fig. 4D and F). Similar results were obtained with DtBRV1 transfectants of CJP4-1D12 (data not shown). Subsequently, the DtBRV1-transfected subisolates JYC1-6T43, -T49, -T50, and -T88 together with the DtBRV1-free parent strain JYC1-6 were also accessed in terms of morphology, growth rate, and virulence. All these DtBRV1-transfected subisolates had morphologies, growth rates, and induced lesions on tea leaves similar to those of JYC1-6 (Fig. 5C through F). Taken together, these results suggest that DtBRV1, with or without dsRNA3, has no or undetectable effects to the biological traits of the host fungus under the conditions investigated, thus causing a latent infection.

## DISCUSSION

In this study, three dsRNAs (dsRNAs 1 to 3) were detected in the avirulent *D. theifolia* strain CJP4-1 and the largest two were identified as the genomic components of a novel botybirnavirus, named DtBRV1, based on sequence alignment and phylogenetic analysis. To date, five botybirnaviruses have been reported in fungi, all infecting filamentous ascomycetes: SsBRV1 and SsBRV2 from *S. sclerotiorum* (20, 21), BpRV1 from *B. porri* (19), and ABV1 and AaBbV1 from *Alternaria* (22, 23). Additionally, soybean leaf-associated botybirnavirus 1 (SlaBRVl) was discovered from a metatranscriptomics survey of soybean phyllosphere phytobiomes (24), while the sequence of Bipolaris maydis botybirnavirus 1 (BmBRV1) from *B. maydis* has been deposited in NCBI (accession no. MF034086-7). To our knowledge, this is the first report of a botybirnavirus infecting *D. theifolia*.



DtBRV1 possesses some molecular traits typical for members of genus *Botybirnavirus*: (i) it forms isometric viral particles *ca*. 40 nm in diameter, within the range of 35–40 nm reported for other botybirnaviruses; (ii) its genome comprises two dsRNA segments of similar sizes; (iii) each genomic segment contains a single large ORF, with an RdRp domain and a proline-rich region in one of them; and (iv) its dsRNAs have a long conserved 5′-UTR and a short 3′-UTR. Moreover, DtBRV1 dsRNAs 1 and 2 share a high similarity of 42.12% over a 433-bp region within their long conserved 5′-UTR, and both their ORFs initiate at the end of the conserved sequence (Fig. 2B). The latter molecular trait has been noted in botybirnaviruses and in Rosellinia necatrix megabirnavirus 1 (RnMBVl) but is rarely observed in other bipartite or multicomponent dsRNA viruses (25).

A dsRNA fragment 1.7 kb in size was previously discovered infecting *S. sclerotiorum* together with SsBRV1 and was considered a satellite RNA since it could be co-precipitated with SsBRV1 (20). In our case, a dsRNA fragment 727 bp in size was detected in association with DtBRV1 (Fig. 3A). This dsRNA3 was detected in the same sucrose gradient fractions following ultracentrifugation, co-transmitted vertically *via* conidiation, and co-transfected into DtBRV1-free strains together with dsRNAs 1 and 2 but not independently transmitted. These results suggest that dsRNA3 is encapsidated in DtBRV1 as an unnecessary component, supporting the notion that it is a satellite dsRNA. Moreover, the full sequence of dsRNA3 shares a considerably high similarity (approximately 40%) with the 5′-terminal regions of dsRNAs 1 and 2. This feature differentiates dsRNA3 from typical satellites that generally have no or little sequence homology with their helper virus and indicates that it most likely originated from a deficient dsRNA component derived from the viral genome. Another striking observation is that dsRNA3 contains a duplicated region encoding a putative peptide. These results indicate that dsRNA3 is a unique satellite dsRNA.

The DtBRVl genome is encapsidated in isometric particles as visualized by TEM following ultracentrifugation. Two predominant protein bands (p65 and p75) were observed by SDS-PAGE, and we concluded that p65 and p75 are two major capsid proteins. By PMF-MS analysis, p65- and p75-generated peptides were shown to be nearly identical and matching partial sequences of both ORF1- and ORF2-encoding proteins, mainly in their N-terminal regions and mostly away from the RdRp domain, as reported for other botybirnaviruses. Each observed band may include two distinct polypeptide species of similar size, one encoded from ORF1 and another encoded from ORF2. This suggests that p75 and p65 are derived from the N-terminal regions of ORF1- and ORF2-encoding polyproteins (217 and 198 kDa, respectively) after processing steps including cleavage. CP generation from a large polyprotein through cleavage is commonly observed in viruses that have a large genome, e.g., Ustilago maydis virus (26) and bursal disease virus (27, 28). The p65 protein(s) may be a degradation product of p75 or derived from p75 *via* a post-translational process, similar to Helminthosporium victoriae virus 190S (19 – 21, 29). The exact number of major CP species in genus *Botybirnavirus* is still not known but may range from three (19 – 21), four (22), or five (23), although it is still unclear how many of the observed bands are degraded or cleaved polypeptides that do not participate in capsid formation. Commonly, formation of an icosahedral T = 1 capsid consisting of 60 copies of one single polypeptide is noted for some typical members of the closely related family *Chrysoviridae*, e.g., Penicillium chrysogenum virus (PcV) (30) and Cryphonectria nitschkei virus 1 (CnV1) (31), while Botryosphaeria dothidea chrysovirus 1 (BdCV1), Botrytis porri RNA virus 1 (19), and members of the family *Quadriviridae* (32) are reported to have a T = 1 capsid consisting of 60 heterodimers, and Magnaporthe oryzae chrysovirus 1 (MoCV1) has multiple CP components (33, 34).

To investigate the biological traits related to DtBRV1 infection, vertical and horizontal transmission experiments were performed with CJP4-1 single-conidium subisolates and dual cultures, respectively, of CJP4-1 with other *D. theifolia* strains. DtBRV1 vertical transmission was shown to be efficient, while horizontal transmission was not observed. The latter might be due to vegetative incompatibility between donor CJP4-1 and recipient strains. Following elimination of DtBRV1 from CJP4-1, the derived subisolates grew at a similar rate but with more dense mycelia as compared with the parent strain and remained avirulent on tea leaves. Transfection of purified DtBRV1 into CJP4-1D12, from which DtBRV1 was previously eliminated, and into the DtBRV1-free, virulent JYC1-6 strain did not lead to reduced growth or hypovirulence regardless of the presence of dsRNA3. These results suggest that DtBRV1 infection has no obvious effects on the biological traits of *D. theifolia* strains under the conditions tested. Therefore, we speculate that the avirulent traits of CJP4-1 are not related to DtBRV1 infection, and DtBRV1 together with dsRNA3 causes a latent infection in the host fungus. Similarly, AaBbV1 and SsBRV1 had no significant effects on their hosts (20, 23), although BpRV1 and SsBRV2 infections attenuated virulence (19, 21).

Collectively, a bipartite dsRNA mycovirus with a unique satellite was detected in *D. theifolia* isolated form tea plants, an important economic crop cultivated in China, representing the first report of a mycovirus infecting *D. theifolia*. A unique satellite was identified in association with DtBRV1, most likely originating from a deficient dsRNA derived from the viral genome. DtBRV1 was efficiently transmitted vertically but not horizontally. Moreover, DtBRV1 exclusively led to a latent infection in the host fungus. This botybirnavirus, together with its satellite, presents interesting molecular and biological traits that will help us understand mycoviruses better.

## MATERIALS AND METHODS

### Fungal strains

*D. theifolia* strains (CJP4-1, JYC1-6, and JYC1-9) were isolated from tea leaves with brown spot symptoms collected from Zigui county, Hubei province, China, and identified by combining sequence data of internal transcribed spacer (ITS), partial β-tubulin (TUB), partial RNA polymerase II second largest subunit (RPB2), and partial large subunit ribosomal RNA (LUS) gene regions. Strains JYC1-6 and JYC1-9 strains (previously termed JYC-1–6 and JYC-1–9, respectively) have been characterized as the etiology of tea leaf brown-black spot disease (18).

### dsRNA extraction

Viral dsRNA was extracted from fungal mycelia using a silica spin column-based method as previously described (35). Fungal mycelium discs (5 mm in diameter) were cultured on PDA plates covered with sterilized cellophane for 5 days. Approximately 0.3 g of fresh mycelium was ground into powder in liquid nitrogen for dsRNA extraction. dsRNA was treated by enzymatic digestion with RNase-free DNaseI and S1 nuclease (New England BioLabs) to remove DNA and ssRNA remains. The obtained dsRNAs were fractionated by 1% (wt/vol) agarose gel electrophoresis, stained with ethidium bromide (0.1 mg/mL), and then visualized by ultraviolet (UV) transillumination.

### NGS sequencing, terminal sequence determination, and RT-PCR amplification

The fungal mycelia of the strain CJP4-1 were collected and then subjected to long non-coding RNA sequencing (lncRNA-seq) in Personal Biotechnology Co. Ltd. (Shanghai, China). The resulting raw data were manipulated by removing low-quality bases, N bases, polyA tails, or residual adapters and assembled using iterative De Bruijn Graph *De Novo* Assembler with Highly Uneven Sequencing Depth (IDBA-UD) (36). Obtained manipulated reads were assembled *de novo* using Velvet with a k-mer value of 17 (37) and CAP3 with default values (38), and then, the contigs were assembled using Vector NTI Advance (version 11.5) software.

Terminal cDNA sequences of dsRNAs were amplified using the rapid amplification of cDNA ends (RACE) protocol as previously described (39). Briefly, the 3′ terminus of each strand of dsRNA was ligated with PC3-T7loop oligo (Table S1) using T4 RNA ligase (TaKaRa, China) at 16°C for 16 h. The oligonucleotide-ligated dsRNA was purified, denatured in DMSO, and subsequently reverse transcribed using M-MLV reverse transcriptase and 3 pmol of PC2. cDNA products were subjected to PCR amplification using sets of specific forward primer/PC2 and PC2/specific reverse primer (Table S1) to amplify the 3′- and 5′-termini, respectively. The PCR amplicons were purified, cloned into pMD18-T vector (TaKaRa, China), and sequenced by Tsingke Biotechnology Co. Ltd. (Wuhan, China). Every nucleotide was determined with at least three independent overlapping clones in both orientations. Sequence assemblies from both cDNA and RACE clones were manipulated using Vector NTI Advance (version 11.5) software to obtain the complete sequence of the viral dsRNA extracted from CJP4-1.

### Phylogenetic and sequence analyses

The full-length cDNA sequences of the dsRNAs were used to predict putative ORFs and the corresponding amino acid sequences using ORF Finder (https://www.ncbi.nlm.nih.gov/orffinder/). The conserved structural domains of the viral proteins were identified using the Protein Family (PFAM; http://genome.jp/tools/motif/). Searches for homologous sequences were performed using the basic local alignment search tool (BLAST) and the National Centre for Biotechnology Information (NCBI) database, whereas the high-similarity mycoviral protein sequences were imported into PhyloSuite (version 1.2.2) for subsequent multiple sequence matching and phylogenetic analysis (40). Multiple alignments of nucleic acid or amino acid sequences were performed with MAFFT (version 7.313), and the outcome was visualized in GeneDoc (http://www.nrbsc.org/downloads). Maximum-likelihood phylogenetic trees with 1,000 ultrafast (41) bootstraps replicates were constructed using IQ-TREE (42) under the model automatically selected by IQ-TREE ('auto' option), as well as the Shimodaira-Hasegawa-like approximate-likelihood ratio tests were used (43). Visualization and refinement of the phylogenetic tree were performed on ITOL (https://itol.embl.de/) (44).

### Viral purification and polypeptide mass fingerprint-mass spectrometry analysis

Viral particle extraction and purification were performed as previously described (13) with some modifications. Briefly, the fungus was grown on sterilized cellophane placed on PDA at 25°C for 7 days, and 36 g of mycelial was harvested, ground in liquid nitrogen, transferred into new tubes containing 3 volumes of 0.1 mol/L phosphate buffer (PB, 8.0 mmol/L Na_2_HPO_4_, 2.0 mmol/L NaH_2_PO_4_, pH 7.0) and 0.5% (vol/vol) β-mercaptoethanol, and mixed gently. The samples were initially centrifuged at 12,000 rpm for 15 min and subsequently ultracentrifuged at 32,000 rpm for 3 hours twice (Optima LE-80K, Backman Coulter Inc. USA). The obtained sediment was resuspended in 0.1 mol/L PB and then ultracentrifuged in sucrose density gradients of 10%–50% (wt/vol) (with an increment of 10%) at 32,000 rpm for 3 h. Following sucrose gradient ultracentrifugation, each sucrose fraction was collected, diluted, and subjected again to ultracentrifugation to pellet virus particles, which were resuspended in 150 µL of 0.1 mol/L PB (pH 7.0). Nucleic acids from each sucrose fraction were extracted using phenol/chloroform and analyzed by 1% (wt/vol) agarose gel electrophoresis to detect viral dsRNA. Moreover, each sucrose fraction was adsorbed on copper grids for 3 min, dried on filter paper for 2 min, stained with 2% (wt/vol) phosphotungstic acid (PTA) for 3 min, and observed under the transmission electron microscope (H7650; Hitachi). Virion sizes were measured with ImageJ. Finally, 200 µL from each sucrose fraction was subjected to protein extraction, analyzed by 12% (wt/vol) SDS-PAGE with 25 mM Tris/glycine and 0.1% SDS, and stained with Coomassie brilliant blue R-250 (Bio-Safe CBB; Bio-Rad, USA). Obtained protein bands were individually excised and subjected to PMF analysis at Sangon Biotech (Shanghai) Co. Ltd., China.

### Mycovirus vertical and horizontal transmission

Mycovirus vertical transmission of the DtBRV1 was accessed *via* conidia as previously described (45). Fungal mycelia were inoculated on alfalfa sticks and incubated on PDA plates for conidium formation at 25°C in darkness for 15 days. The resulting conidia were diluted into the concentrations of 10^4^–10^5^ conidia/mL, and 100 µL of the conidial suspension was spread onto PDA and incubated at 25°C in darkness for colony formation. The colonies generated from conidia were transferred on fresh PDA plates for further growth and dsRNA extraction.

Mycovirus horizontal transmission of was accessed *via* hyphal anastomosis as previously described (13). Briefly, each donor strain was dually cultured with a recipient strain on PDA plates at 25°C for 7–10 days to allow contact between the two colonies. After incubation of the contact cultures, mycelial agar plugs were excised from the colony margin of the recipient strain (as far as possible from the donor colony) and transferred on fresh PDA plates for further growth and dsRNA extraction. Each contact culture experiment was conducted in triplicate: in total, five subisolates were derived from each recipient strain and 15 mycelium plugs were obtained from the contact cultures.

### Protoplast transfection with virions

Fungal protoplasts were isolated and transfected with purified viral particles as previously described (46). Protoplasts were isolated from actively growing mycelia of the virus-free *D. theifolia* strains CJP4-1D12 and JYC1-6 and transfected with 70–80 μg virions in the presence of PEG 6000. The transfected protoplast suspension was incubated without shaking at 25°C in darkness for 16 hours and spread on bottom agar (BA) plates containing 0.3% yeast extract, 0.3% casein hydrolysate, 20% sucrose, and 1% agar. Mycelial agar plugs generated from the protoplasts were transferred on fresh PDA plates for further growth and dsRNA extraction.

### Growth rate, morphology, and virulence assays

Fungal growth rate and morphology were assessed in triplicate for each strain or subisolate as previously described (13). Fungal virulence was determined in triplicate for each strain or subisolate following inoculation of detached, wounded tea leaves (*C. sinensis* var. E’cha no.1) as previously described (13). At 5 dpi, lesions that had developed on the inoculated leaves were measured and photographed.

## Data Availability

The complete nucleotide sequence of DtBRV1 reported in this article has been deposited in the GenBank database under accession numbers OQ078673 to OQ078675.

## SUPPLEMENTAL MATERIAL

Supplemental material spectrum.00033-23-s0001.pdf Fig. S1 to S5; Tables S1 to S3.

ASM does not own the copyrights to Supplemental Material that may be linked to, or accessed through, an article. The authors have granted ASM a non-exclusive, world-wide license to publish the Supplemental Material files. Please contact the corresponding author directly for reuse.
