# Supplementary material for: A novel botybirnavirus with a unique satellite dsRNA causes latent infection in Didymella theifolia isolated from tea plants
Source: Microbiol Spectr. 2023 Nov 14;11(6):e00033-23. doi: 10.1128/spectrum.00033-23 (PMC10714997; doi:10.1128/spectrum.00033-23)
Supplement: Supplemental material — Fig. S1 to S5; Tables S1 to S3. [file spectrum.00033-23-s0001.pdf]

Fig. S1 Alignment respectively of the (A) 5'-terminal and (B) 3'-terminal sequences of the coding strands of dsRNAs 1 and 2. Identical nucleotides are highlighted with dark blue color.

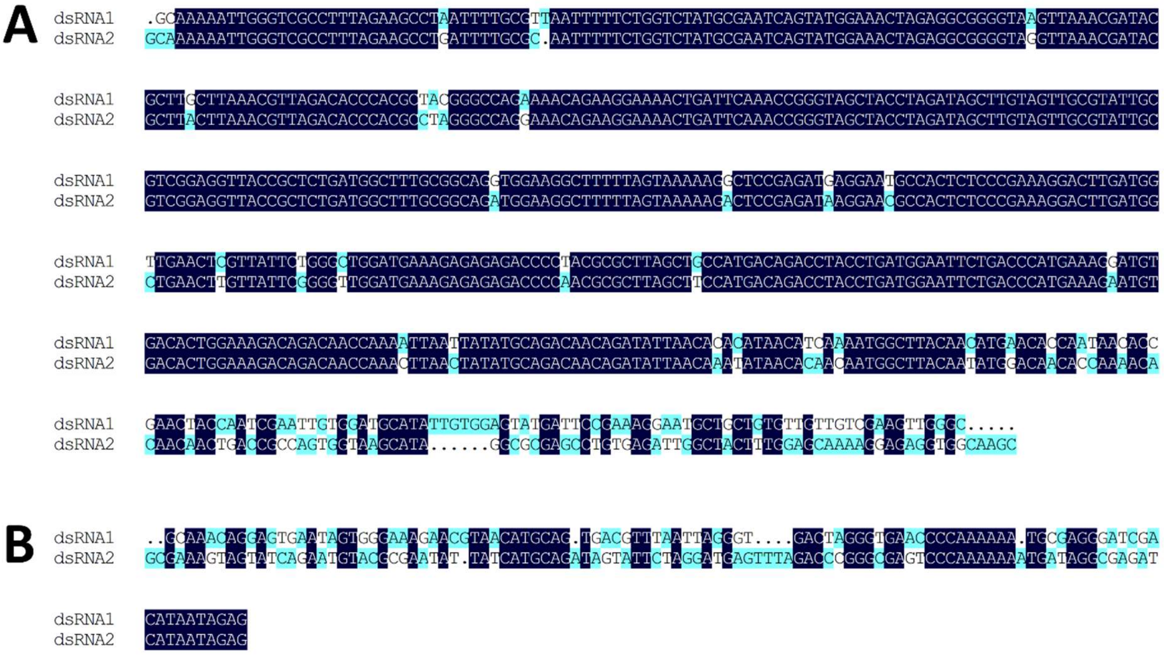

Fig. S2 Multiple alignment of RdRp sequences of DtBRV1 and other RNA viruses. Lines above the aligned sequences indicate to the positions of the eight motifs (I to VIII). Number between each two motifs represented the number of amino acid residues separating the two motifs. Identical, high, and low conserved residues are indicated as red (100% identity), pink (80%), yellow (60%), respectively.

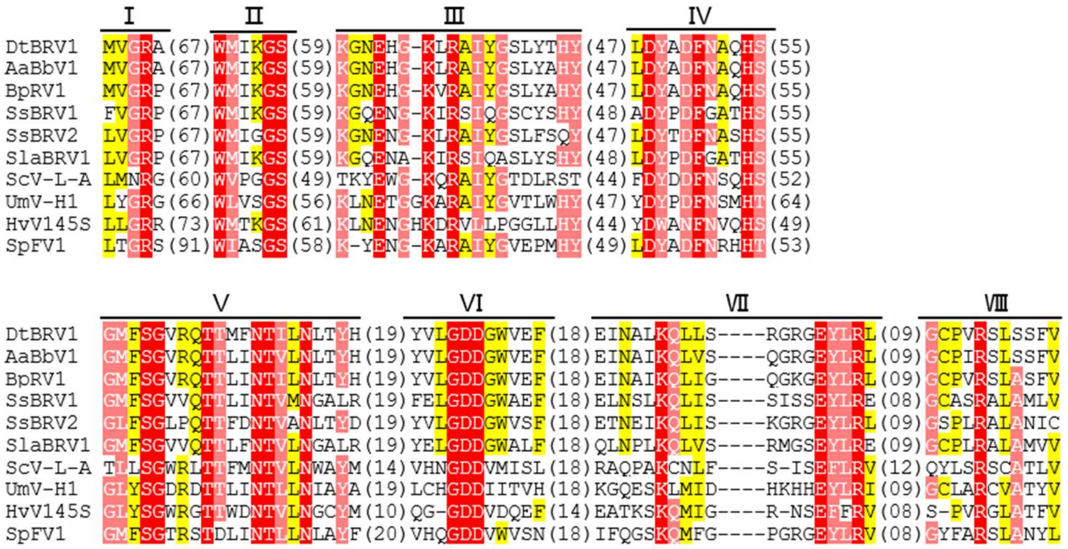

Fig. S3 Multiplex RT-PCR of dsRNAs 2 and 3 based on the nucleic acids extracted from 10% to 50% sucrose fractions at serial dilutions (0, 30 and 540 folds). Strain CJP4-1 and JYC1-6 were involved as a positive and a negative control respectively, while the ddH_2O was used as blank control. The expected PCR product is 390 bp in size for dsRNA2, and both 313 and 578 bp for dsRNA3.

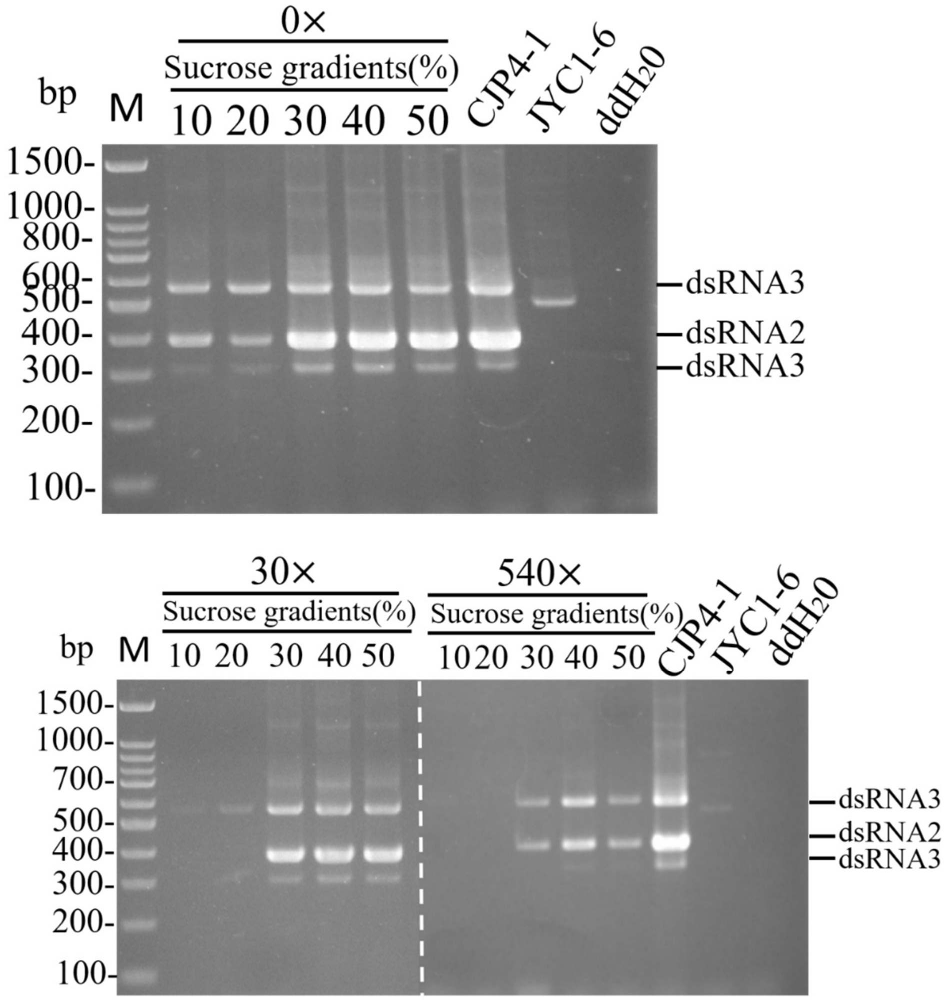

Fig. S4 Horizontal transmission of DtBRV1. (A) Transmission of dsRNA from strain CJP-4-1 to virulent strain JYC-1-6 and JYC-1-9. Strain CJP-4-1 and JYC-1-6 were co-cultured for 10 days (left). Strain CJP-4-1 and JYC-1-9 were co-cultured for 7 days (right). (B) Detection of confrontation culture of strain CJP-4-1, JYC-1-6and JYC-1-9.

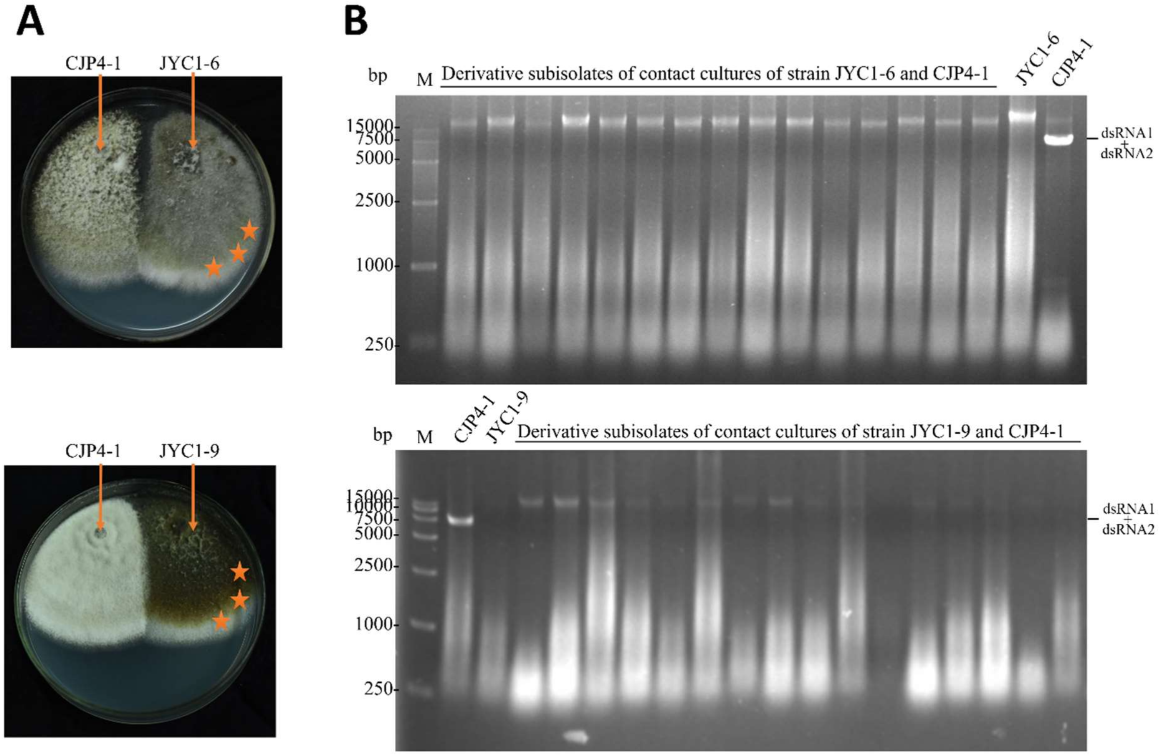

Fig. S5 Protoplast transfection. (A) Agarose gel electrophoresis of dsRNA extracted from mycelia of generated protoplasts CJP4-1D12-T1 to CJP4-1D12-T48 and (B) JYC1-6T1 to JYC1-6T107 (Lanes 1 to 107) following transfection of CJP4-1D12 and JYC-1-6 with DtBRV1. CJP4-1 and JYC1-6 were respectively used as positive and negative controls.

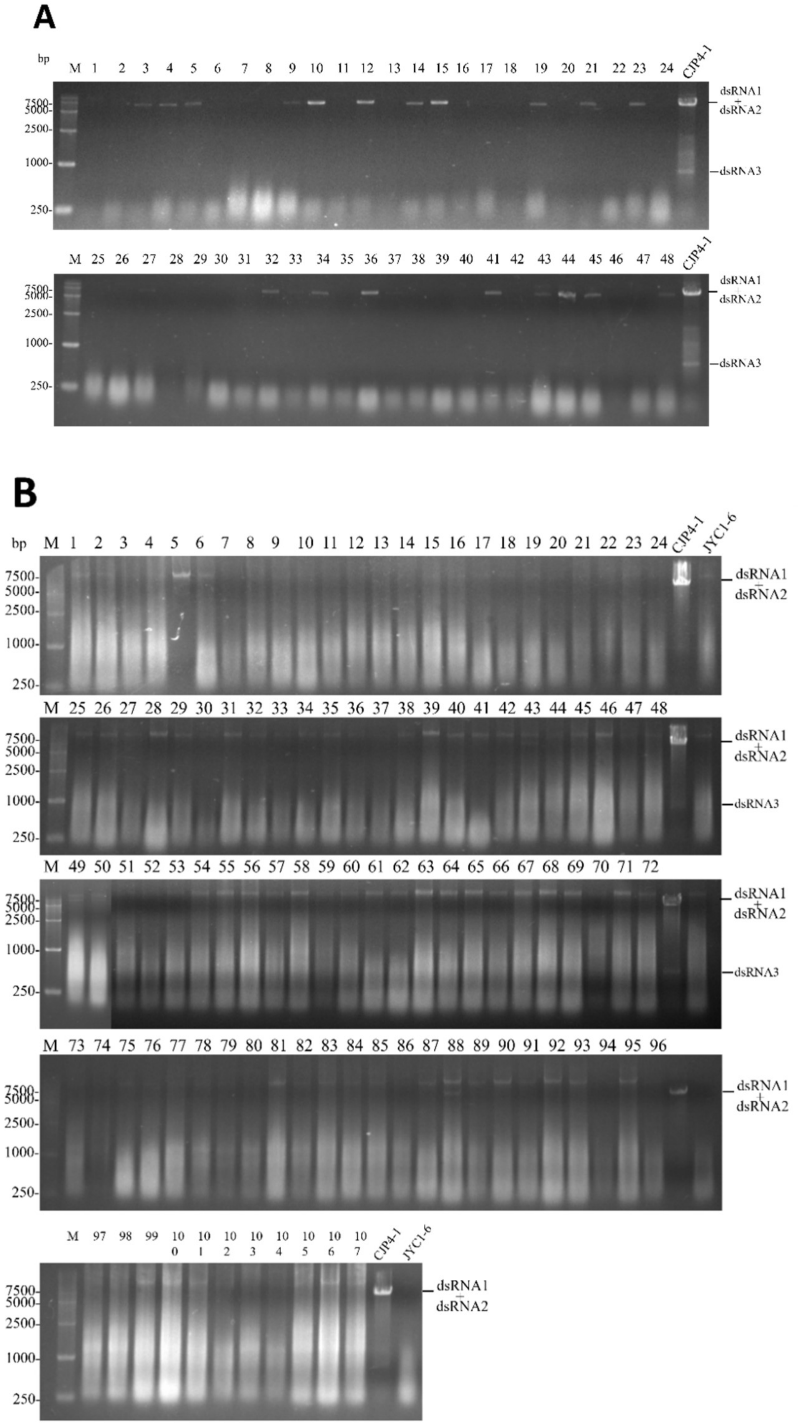

Table S1 Primers and adaptors used in virus amplification.

Primer/Adaptor	Primer sequence (5'→3')
PC3-T7-loop adapter	p-GGATCCCGGGAATTCGGTAATACGACTCACTATATTTTTATAGTGAGTCGTATTA-OH
PC2 primer	CCGAATTCCCGGGATCC
DtBRV1-F1	AACAACTAGCCAATACCTTCG
DtBRV1-R1	CTTCCTCTATGCCAGCGTTC
dsRNA3-F1	CCGCCTTTATATCATACCCTG
dsRNA3-R1	CGCAAAAATTAGCACTCCAAC
DtBRV1-dsRNA1-R	GGCAAACATACACGTACTGG
DtBRV1-dsRNA1-F	ACAGAACAACAGCACACGAG
DtBRV1-dsRNA2-R	ACCATCAGCAAACCGTATCG
DtBRV1-dsRNA2-F	GCGATAAGAAGAGAAGCCAC
M13F-47	CGCCAGGGTTTTCCCAGTCACGAC
M13R-48	AGCGGATAACAATTTCACACAGGA

Table S2 Peptide mass fingerprinting analysis of p75.

Putative ORF	Start-End	Observed	Mr(expt)	Mr(calc)	pp m	M iss	Sequence
ORF1	267-281	823.3593	1644.704	1644.7016	1	0	QTDPAAQPEENSGYT
ORF1	267-281	831.8723	1661.73	1661.7281	1	0	QTDPAAQPEENSGYT
ORF1	267-281	831.8725	1661.7304	1661.7281	1	0	QTDPAAQPEENSGYT
ORF1	267-281	554.9175	1661.7308	1661.7281	2	0	QTDPAAQPEENSGYT
ORF1	267-281	832.3643	1662.7141	1662.7121	1	0	QTDPAAQPEENSGYT
ORF1	282-290	479.7507	957.4869	957.4879	-1	0	TAPAISNES
ORF1	282-290	479.7514	957.4882	957.4879	0	0	TAPAISNES
ORF1	282-290	480.2421	958.4696	958.4719	-2	0	TAPAISNES
ORF1	291-324	1760.8274	3519.6402	3519.6331	2	0	SGEPVDAPAHTGWLSWEDGSLGGNATYYA GSIVS
ORF1	291-324	1174.2238	3519.6497	3519.6331	5	0	SGEPVDAPAHTGWLSWEDGSLGGNATYYA GSIVS
ORF1	291-324	880.9201	3519.6511	3519.6331	5	0	SGEPVDAPAHTGWLSWEDGSLGGNATYYA GSIVS
ORF1	291-324	705.137	3520.6484	3520.6171	9	0	SGEPVDAPAHTGWLSWEDGSLGGNATYYA GSIVS
ORF1	325-342	740.3467	2218.0184	2218.0192	0	0	STYEHTWNQHLQANFNTK
ORF1	325-342	555.5126	2218.0214	2218.0192	1	0	STYEHTWNQHLQANFNTK
ORF1	325-342	740.348	2218.0222	2218.0192	1	0	STYEHTWNQHLQANFNTK
ORF1	325-342	1110.0185	2218.0225	2218.0192	2	0	STYEHTWNQHLQANFNTK
ORF1	325-342	1110.5111	2219.0077	2219.0032	2	0	STYEHTWNQHLQANFNTK
ORF1	325-342	740.6768	2219.0087	2219.0032	2	0	STYEHTWNQHLQANFNTK
ORF1	325-342	555.7611	2219.0153	2219.0032	5	0	STYEHTWNQHLQANFNTK
ORF1	325-354	1187.8995	3560.6768	3560.6709	2	1	STYEHTWNQHLQANFNTKYPPGSTIEQNVS
ORF1	325-354	713.343	3561.6787	3561.6549	7	1	STYEHTWNQHLQANFNTKYPPGSTIEQNVS
ORF1	325-354	891.4282	3561.6835	3561.6549	8	1	STYEHTWNQHLQANFNTKYPPGSTIEQNVS
ORF1	343-354	454.2335	1359.6788	1359.6783	0	0	KPPGSTIEQNVS
ORF1	343-354	680.848	1359.6815	1359.6783	2	0	KPPGSTIEQNVS
ORF1	355-383	805.8978	3219.5623	3219.5474	5	0	SMSLLLTPQFTADYFWGYGAPSPEAEITA
ORF1	355-383	1074.1951	3219.5634	3219.5474	5	0	SMSLLLTPQFTADYFWGYGAPSPEAEITA
ORF1	355-383	1079.5263	3235.557	3235.5424	5	0	SMSLLLTPQFTADYFWGYGAPSPEAEITA
ORF1	384-400	911.9621	1821.9096	1821.9084	1	0	AGFVDSFTVVQMTHVGK
ORF1	384-400	911.9622	1821.9098	1821.9084	1	0	AGFVDSFTVVQMTHVGK
ORF1	384-400	608.3112	1821.9118	1821.9084	2	0	AGFVDSFTVVQMTHVGK
ORF1	384-400	608.3114	1821.9124	1821.9084	2	0	AGFVDSFTVVQMTHVGK
ORF1	384-400	608.3116	1821.913	1821.9084	3	0	AGFVDSFTVVQMTHVGK
ORF1	384-400	608.3128	1821.9167	1821.9084	5	0	AGFVDSFTVVQMTHVGK
ORF1	384-400	608.3132	1821.9178	1821.9084	5	0	AGFVDSFTVVQMTHVGK
ORF1	384-400	608.3151	1821.9233	1821.9084	8	0	AGFVDSFTVVQMTHVGK
ORF1	384-400	613.6424	1837.9055	1837.9033	1	0	AGFVDSFTVVQMTHVGK
ORF1	384-400	613.6432	1837.9079	1837.9033	2	0	AGFVDSFTVVQMTHVGK
ORF1	384-400	613.6435	1837.9087	1837.9033	3	0	AGFVDSFTVVQMTHVGK
ORF1	384-400	613.644	1837.9101	1837.9033	4	0	AGFVDSFTVVQMTHVGK
ORF1	384-400	919.9641	1837.9137	1837.9033	6	0	AGFVDSFTVVQMTHVGK
ORF1	401-408	449.7901	897.5656	897.5647	1	0	KILIPVSE
ORF1	401-408	449.7902	897.5658	897.5647	1	0	KILIPVSE
ORF1	401-408	449.7907	897.5668	897.5647	2	0	KILIPVSE
ORF1	401-408	449.791	897.5674	897.5647	3	0	KILIPVSE
ORF1	401-408	449.7912	897.5678	897.5647	3	0	KILIPVSE
ORF1	409-424	914.9699	1827.9252	1827.9155	5	0	ETIGYNVPFGLSVYNT
ORF1	409-424	610.3167	1827.9282	1827.9155	7	0	ETIGYNVPFGLSVYNT
ORF1	425-445	776.0657	2325.1753	2325.1675	3	0	TMPFNSLASITPTSDLYAQIH

ORF1	425-445	776.3937	2326.1593	2326.1515	3	0	TMPFNSLASITPTSDLYAQIH
ORF1	425-445	1164.0903	2326.1661	2326.1515	6	0	TMPFNSLASITPTSDLYAQIH
ORF1	425-445	781.3996	2341.1771	2341.1624	6	0	TMPFNSLASITPTSDLYAQIH
ORF1	425-445	1171.5963	2341.1781	2341.1624	7	0	TMPFNSLASITPTSDLYAQIH
ORF1	425-445	781.7288	2342.1645	2342.1464	8	0	TMPFNSLASITPTSDLYAQIH
ORF1	448-455	517.2568	1032.4991	1032.4989	0	1	KVTDDYHM
ORF1	449-455	453.2086	904.4026	904.4039	-1	0	VTDDYHM
ORF1	456-463	460.2551	918.4956	918.4957	0	0	MILTASQG
ORF1	456-463	468.2524	934.4903	934.4906	0	0	MILTASQG
ORF1	456-463	468.2532	934.4919	934.4906	1	0	MILTASQG
ORF1	464-477	774.8755	1547.7364	1547.7337	2	0	GNNHIALGATMYML
ORF1	464-477	516.9195	1547.7367	1547.7337	2	0	GNNHIALGATMYML
ORF1	464-477	387.9415	1547.7368	1547.7337	2	0	GNNHIALGATMYML
ORF1	464-477	516.9195	1547.7368	1547.7337	2	0	GNNHIALGATMYML
ORF1	464-477	516.9199	1547.7377	1547.7337	3	0	GNNHIALGATMYML
ORF1	464-477	516.9203	1547.739	1547.7337	3	0	GNNHIALGATMYML
ORF1	464-477	516.9214	1547.7424	1547.7337	6	0	GNNHIALGATMYML
ORF1	464-477	517.2487	1548.7243	1548.7177	4	0	GNNHIALGATMYML
ORF1	464-477	522.2508	1563.7306	1563.7286	1	0	GNNHIALGATMYML
ORF1	464-477	782.8733	1563.7321	1563.7286	2	0	GNNHIALGATMYML
ORF1	464-477	522.5784	1564.7134	1564.7126	0	0	GNNHIALGATMYML
ORF1	464-477	522.5788	1564.7145	1564.7126	1	0	GNNHIALGATMYML
ORF1	464-477	783.3667	1564.7188	1564.7126	4	0	GNNHIALGATMYML
ORF1	464-477	527.5812	1579.7217	1579.7235	-1	0	GNNHIALGATMYML
ORF1	464-477	790.8688	1579.7231	1579.7235	0	0	GNNHIALGATMYML
ORF1	464-477	527.9104	1580.7095	1580.7075	1	0	GNNHIALGATMYML
ORF1	464-477	791.3627	1580.7109	1580.7075	2	0	GNNHIALGATMYML
ORF1	483-502	731.6931	2192.0574	2192.0498	3	0	VAQEQGANPFITVESEGFNF
ORF1	483-502	1097.5287	2193.0428	2193.0338	4	0	VAQEQGANPFITVESEGFNF
ORF1	483-502	1097.5343	2193.0541	2193.0338	9	0	VAQEQGANPFITVESEGFNF
ORF1	503-519	846.4528	1690.891	1690.889	1	0	FLGAVGSADSVLVETAT
ORF1	503-519	564.6377	1690.8913	1690.889	1	0	FLGAVGSADSVLVETAT
ORF1	503-519	846.4533	1690.892	1690.889	2	0	FLGAVGSADSVLVETAT
ORF1	503-519	564.6382	1690.8929	1690.889	2	0	FLGAVGSADSVLVETAT
ORF1	503-519	846.4538	1690.8931	1690.889	2	0	FLGAVGSADSVLVETAT
ORF1	503-519	564.6385	1690.8936	1690.889	3	0	FLGAVGSADSVLVETAT
ORF1	503-519	846.4543	1690.8941	1690.889	3	0	FLGAVGSADSVLVETAT
ORF1	503-519	846.4545	1690.8944	1690.889	3	0	FLGAVGSADSVLVETAT
ORF1	503-519	564.6395	1690.8968	1690.889	5	0	FLGAVGSADSVLVETAT
ORF1	503-519	564.6396	1690.8968	1690.889	5	0	FLGAVGSADSVLVETAT
ORF1	520-534	841.8959	1681.7773	1681.7626	9	0	TFAANTPMNMVTMPN
ORF1	520-534	849.3946	1696.7746	1696.7735	1	0	TFAANTPMNMVTMPN
ORF1	520-534	566.599	1696.7752	1696.7735	1	0	TFAANTPMNMVTMPN
ORF1	520-534	849.3953	1696.776	1696.7735	1	0	TFAANTPMNMVTMPN
ORF1	520-534	566.5997	1696.7772	1696.7735	2	0	TFAANTPMNMVTMPN
ORF1	520-534	857.3895	1712.7644	1712.7684	-2	0	TFAANTPMNMVTMPN
ORF1	520-534	571.9303	1712.769	1712.7684	0	0	TFAANTPMNMVTMPN
ORF1	520-534	857.393	1712.7714	1712.7684	2	0	TFAANTPMNMVTMPN
ORF1	520-534	857.394	1712.7734	1712.7684	3	0	TFAANTPMNMVTMPN
ORF1	520-534	865.3895	1728.7644	1728.7634	1	0	TFAANTPMNMVTMPN
ORF1	520-534	865.3901	1728.7657	1728.7634	1	0	TFAANTPMNMVTMPN
ORF1	520-534	865.3915	1728.7684	1728.7634	3	0	TFAANTPMNMVTMPN
ORF1	535-552	1124.4943	2246.9739	2246.9725	1	0	NAVEDEHLYMYYLCGNTL
ORF1	535-552	750.0005	2246.9798	2246.9725	3	0	NAVEDEHLYMYYLCGNTL
ORF1	535-552	1124.9908	2247.9671	2247.9565	5	0	NAVEDEHLYMYYLCGNTL
ORF1	535-552	1124.991	2247.9674	2247.9565	5	0	NAVEDEHLYMYYLCGNTL
ORF1	535-552	1132.4949	2262.9752	2262.9674	3	0	NAVEDEHLYMYYLCGNTL
ORF1	535-552	755.3326	2262.9759	2262.9674	4	0	NAVEDEHLYMYYLCGNTL

ORF1	535-552	755.3333	2262.9782	2262.9674	5	0	NAVEDEHLYMYYLCGNTL
ORF1	535-552	755.6602	2263.9588	2263.9514	3	0	NAVEDEHLYMYYLCGNTL
ORF1	535-552	1132.9869	2263.9592	2263.9514	3	0	NAVEDEHLYMYYLCGNTL
ORF1	553-566	551.3031	1650.8875	1650.8842	2	1	LRIPWQQDANSPVD
ORF1	553-566	826.4526	1650.8906	1650.8842	4	1	LRIPWQQDANSPVD
ORF1	555-566	461.574	1381.7003	1381.699	1	0	IPWQQDANSPVD
ORF1	555-566	691.8576	1381.7007	1381.699	1	0	IPWQQDANSPVD
ORF1	555-566	692.3507	1382.6869	1382.683	3	0	IPWQQDANSPVD
ORF1	555-580	1026.171	3075.491	3075.476	5	1	IPWQQDANSPVKDLFSCLDAYVPEQF
ORF1	567-580	856.903	1711.7914	1711.7876	2	0	DLFSCLDAYVPEQF
ORF1	567-580	856.9046	1711.7947	1711.7876	4	0	DLFSCLDAYVPEQF
ORF1	581-591	644.3558	1286.6971	1286.6983	-1	0	FILTPIANDQI
ORF1	581-591	644.3563	1286.6981	1286.6983	0	0	FILTPIANDQI
ORF1	581-591	644.3571	1286.6997	1286.6983	1	0	FILTPIANDQI
ORF1	581-591	429.9074	1286.7003	1286.6983	2	0	FILTPIANDQI
ORF1	581-591	644.3575	1286.7004	1286.6983	2	0	FILTPIANDQI
ORF1	581-591	644.85	1287.6854	1287.6823	2	0	FILTPIANDQI
ORF1	581-601	789.1031	2364.2876	2364.2801	3	1	FILTPIANDQRIEPLPVGTDI
ORF1	581-601	789.4322	2365.2747	2365.2641	4	1	FILTPIANDQRIEPLPVGTDI
ORF1	592-601	366.2051	1095.5934	1095.5924	1	0	IEPLPVGTDI
ORF1	592-601	548.8045	1095.5945	1095.5924	2	0	IEPLPVGTDI
ORF1	592-601	548.8045	1095.5945	1095.5924	2	0	IEPLPVGTDI
ORF1	592-601	548.8061	1095.5976	1095.5924	5	0	IEPLPVGTDI
ORF1	604-616	502.9565	1505.8478	1505.8453	2	1	RVDSYLLETIVAK
ORF1	604-616	753.9315	1505.8485	1505.8453	2	1	RVDSYLLETIVAK
ORF1	605-616	450.9225	1349.7457	1349.7442	1	0	VDSYLLETIVAK
ORF1	605-616	675.8809	1349.7473	1349.7442	2	0	VDSYLLETIVAK
ORF1	605-616	675.8822	1349.7498	1349.7442	4	0	VDSYLLETIVAK
ORF1	605-616	675.8822	1349.7498	1349.7442	4	0	VDSYLLETIVAK
ORF1	617-631	621.6511	1861.9316	1861.9298	1	0	KFTTHNLWGQLPVMA
ORF1	617-631	931.9737	1861.9328	1861.9298	2	0	KFTTHNLWGQLPVMA
ORF1	617-631	939.973	1877.9315	1877.9247	4	0	KFTTHNLWGQLPVMA
ORF1	632-648	598.6756	1793.0051	1792.9988	4	0	AFAWAILAHPATAVNIF
ORF1	632-648	897.5102	1793.0059	1792.9988	4	0	AFAWAILAHPATAVNIF
ORF1	649-670	628.5678	2510.2422	2510.2376	2	0	FPAPMHTSELQLNLWPNTTASV
ORF1	649-670	632.566	2526.2351	2526.2325	1	0	FPAPMHTSELQLNLWPNTTASV
ORF1	649-670	843.087	2526.2392	2526.2325	3	0	FPAPMHTSELQLNLWPNTTASV
ORF1	649-670	843.0895	2526.2466	2526.2325	6	0	FPAPMHTSELQLNLWPNTTASV
ORF1	649-670	843.4204	2527.2394	2527.2165	9	0	FPAPMHTSELQLNLWPNTTASV
ORF1	674-693	1082.5986	2163.1827	2163.18	1	1	ILGDKDHFTSLHVATVIAAN
ORF1	674-693	722.0686	2163.184	2163.18	2	1	ILGDKDHFTSLHVATVIAAN
ORF1	674-693	541.8035	2163.1849	2163.18	2	1	ILGDKDHFTSLHVATVIAAN
ORF1	674-693	541.8053	2163.1923	2163.18	6	1	ILGDKDHFTSLHVATVIAAN
ORF1	694-720	584.8959	2919.4433	2919.4324	4	0	NCEEVLTDAIVSATTAVGIPYTHPAYA
ORF1	694-720	730.8681	2919.4433	2919.4324	4	0	NCEEVLTDAIVSATTAVGIPYTHPAYA
ORF1	694-720	974.1577	2919.4512	2919.4324	6	0	NCEEVLTDAIVSATTAVGIPYTHPAYA
ORF1	694-720	974.4845	2920.4317	2920.4164	5	0	NCEEVLTDAIVSATTAVGIPYTHPAYA
ORF1	694-720	974.4854	2920.4344	2920.4164	6	0	NCEEVLTDAIVSATTAVGIPYTHPAYA
ORF1	721-729	501.7642	1001.5138	1001.5142	0	0	ATVEQELGH
ORF1	721-729	501.7649	1001.5152	1001.5142	1	0	ATVEQELGH
ORF1	721-729	501.7654	1001.5162	1001.5142	2	0	ATVEQELGH
ORF1	773-780	490.2829	978.5513	978.5498	1	1	SLDTKLFA
ORF1	781-804	1356.1478	2710.2811	2710.2762	2	0	ASSYLVVNEEPEGEGWQLVFSENR
ORF1	781-804	904.4345	2710.2818	2710.2762	2	0	ASSYLVVNEEPEGEGWQLVFSENR
ORF1	781-804	1356.1484	2710.2822	2710.2762	2	0	ASSYLVVNEEPEGEGWQLVFSENR
ORF1	781-804	904.4384	2710.2934	2710.2762	6	0	ASSYLVVNEEPEGEGWQLVFSENR
ORF1	781-804	1356.155	2710.2955	2710.2762	7	0	ASSYLVVNEEPEGEGWQLVFSENR
ORF1	781-805	956.4669	2866.3788	2866.3773	1	1	ASSYLVVNEEPEGEGWQLVFSENKK

ORF1	781-805	956.4719	2866.3937	2866.3773	6	1	ASSYLVVNEEPEGEGWQLVFSENKK
ORF1	781-805	717.8534	2867.3845	2867.3613	8	1	ASSYLVVNEEPEGEGWQLVFSENKK
ORF1	781-805	1434.7003	2867.3861	2867.3613	9	1	ASSYLVVNEEPEGEGWQLVFSENKK
ORF1	816-828	752.4138	1502.813	1502.8093	2	1	SREALVATWLTES
ORF1	816-828	501.9451	1502.8136	1502.8093	3	1	SREALVATWLTES
ORF1	818-828	420.8999	1259.678	1259.6761	1	0	EALVATWLTES
ORF1	818-828	630.8469	1259.6792	1259.6761	2	0	EALVATWLTES
ORF1	818-828	630.8478	1259.681	1259.6761	4	0	EALVATWLTES
ORF1	818-828	630.8485	1259.6824	1259.6761	5	0	EALVATWLTES
ORF1	834-862	1569.7706	3137.5267	3137.5227	1	0	ASDITYYQHETAEVDSTVVLPVMLGNTGN
ORF1	834-862	1046.8529	3137.5368	3137.5227	4	0	ASDITYYQHETAEVDSTVVLPVMLGNTGN
ORF1	834-862	1046.8529	3137.5368	3137.5227	4	0	ASDITYYQHETAEVDSTVVLPVMLGNTGN
ORF1	834-862	1046.854	3137.5403	3137.5227	6	0	ASDITYYQHETAEVDSTVVLPVMLGNTGN
ORF1	834-862	1046.8555	3137.5448	3137.5227	7	0	ASDITYYQHETAEVDSTVVLPVMLGNTGN
ORF1	834-862	1570.2753	3138.536	3138.5067	9	0	ASDITYYQHETAEVDSTVVLPVMLGNTGN
ORF1	834-862	789.3879	3153.5223	3153.5176	1	0	ASDITYYQHETAEVDSTVVLPVMLGNTGN
ORF1	834-862	1052.183	3153.5271	3153.5176	3	0	ASDITYYQHETAEVDSTVVLPVMLGNTGN
ORF1	834-862	1052.1871	3153.5394	3153.5176	7	0	ASDITYYQHETAEVDSTVVLPVMLGNTGN
ORF1	865-880	650.3525	1948.0356	1948.0319	2	0	LTNLAGRPQLWDYFVG
ORF1	865-880	975.0258	1948.0371	1948.0319	3	0	LTNLAGRPQLWDYFVG
ORF1	865-880	650.3532	1948.0377	1948.0319	3	0	LTNLAGRPQLWDYFVG
ORF1	865-880	650.685	1949.033	1949.0159	9	0	LTNLAGRPQLWDYFVG
ORF1	881-888	365.7267	729.4388	729.4385	0	0	GLTGLAAS
ORF1	881-888	365.727	729.4395	729.4385	1	0	GLTGLAAS
ORF1	889-897	449.257	896.4995	896.4967	3	0	SVVPPAAEN
ORF1	1272-1279	436.2441	870.4737	870.4745	-1	0	IPAGQVMS
ORF1	1343-1351	527.3129	1052.6113	1052.6091	2	0	ELNVAVRPA
ORF1	1425-1436	701.864	1401.7135	1401.7099	3	0	DNSVLLEESLQS
ORF1	1478-1487	591.2952	1180.5759	1180.5764	0	0	GFVPTEEFQI
ORF1	1707-1718	622.3188	1242.623	1242.6204	2	0	DSLAGQPEAVTS
ORF1	1752-1758	455.7381	909.4617	909.4596	2	0	EYSVVWH
ORF1	1782-1791	556.8024	1111.5902	1111.5873	3	0	TTAHEELLAV
ORF1	1825-1830	431.7261	861.4377	861.4457	-9	2	QYRKQI
ORF2	668-690	1239.599	2477.1834	2477.1744	4	0	DLALTTNAGIEEAHAFMSETISG
ORF2	668-690	826.7382	2477.1928	2477.1744	7	0	DLALTTNAGIEEAHAFMSETISG
ORF2	668-690	831.7352	2492.1838	2492.1853	-1	0	DLALTTNAGIEEAHAFMSETISG
ORF2	668-690	831.7362	2492.1868	2492.1853	1	0	DLALTTNAGIEEAHAFMSETISG
ORF2	668-690	831.737	2492.1892	2492.1853	2	0	DLALTTNAGIEEAHAFMSETISG
ORF2	668-690	831.7379	2492.1919	2492.1853	3	0	DLALTTNAGIEEAHAFMSETISG
ORF2	668-690	831.7382	2492.1928	2492.1853	3	0	DLALTTNAGIEEAHAFMSETISG
ORF2	668-690	831.7383	2492.1931	2492.1853	3	0	DLALTTNAGIEEAHAFMSETISG
ORF2	668-690	1247.1047	2492.1949	2492.1853	4	0	DLALTTNAGIEEAHAFMSETISG
ORF2	668-690	831.7395	2492.1966	2492.1853	5	0	DLALTTNAGIEEAHAFMSETISG
ORF2	668-690	831.7397	2492.1971	2492.1853	5	0	DLALTTNAGIEEAHAFMSETISG
ORF2	668-690	1247.5985	2493.1825	2493.1693	5	0	DLALTTNAGIEEAHAFMSETISG
ORF2	668-690	1247.6018	2493.1891	2493.1693	8	0	DLALTTNAGIEEAHAFMSETISG
ORF2	691-707	640.9899	1919.948	1919.9418	3	0	GQSQLISAWATYTFGYS
ORF2	691-707	960.9813	1919.9481	1919.9418	3	0	GQSQLISAWATYTFGYS
ORF2	708-727	544.8079	2175.2024	2175.2011	1	0	SVELQQALNLDASHLVGLID
ORF2	708-727	1088.6095	2175.2045	2175.2011	2	0	SVELQQALNLDASHLVGLID
ORF2	708-727	726.0761	2175.2066	2175.2011	3	0	SVELQQALNLDASHLVGLID
ORF2	708-727	726.4059	2176.1959	2176.1851	5	0	SVELQQALNLDASHLVGLID
ORF2	728-761	723.3916	3611.9216	3611.8869	10	0	DTLATGVLRPASLLIANTTIAQGPISMVWDE TTN
ORF2	728-761	1205.3043	3612.8911	3612.8709	6	0	DTLATGVLRPASLLIANTTIAQGPISMVWDE TTN
ORF2	728-761	1814.4636	3626.9126	3626.8978	4	0	DTLATGVLRPASLLIANTTIAQGPISMVWDE TTN

ORF2	728-761	1209.9791	3626.9154	3626.8978	5	0	DTLATGVLRPASLLIANTTIAQGPISMVWDE TTN
ORF2	728-761	726.3909	3626.918	3626.8978	6	0	DTLATGVLRPASLLIANTTIAQGPISMVWDE TTN
ORF2	764-776	464.5901	1390.7484	1390.7456	2	1	EAAKIYATNPVSM
ORF2	764-776	696.3837	1390.7529	1390.7456	5	1	EAAKIYATNPVSM
ORF2	768-776	496.7729	991.5313	991.5338	-3	0	IYATNPVSM
ORF2	768-776	496.7745	991.5345	991.5338	1	0	IYATNPVSM
ORF2	768-776	496.7746	991.5347	991.5338	1	0	IYATNPVSM
ORF2	768-776	496.7758	991.537	991.5338	3	0	IYATNPVSM
ORF2	768-776	496.7765	991.5384	991.5338	5	0	IYATNPVSM
ORF2	777-790	582.9656	1745.8749	1745.8698	3	0	MLTYIYSNEPQIFK
ORF2	777-790	873.9475	1745.8804	1745.8698	6	0	MLTYIYSNEPQIFK
ORF2	777-790	881.9411	1761.8676	1761.8647	2	0	MLTYIYSNEPQIFK
ORF2	777-790	881.9417	1761.8688	1761.8647	2	0	MLTYIYSNEPQIFK
ORF2	777-790	588.2975	1761.8706	1761.8647	3	0	MLTYIYSNEPQIFK
ORF2	791-802	650.3486	1298.6827	1298.6805	2	0	KHGPLLANFAMV
ORF2	791-802	650.3487	1298.6828	1298.6805	2	0	KHGPLLANFAMV
ORF2	791-802	433.9019	1298.684	1298.6805	3	0	KHGPLLANFAMV
ORF2	791-802	433.9025	1298.6858	1298.6805	4	0	KHGPLLANFAMV
ORF2	791-802	658.3454	1314.6763	1314.6754	1	0	KHGPLLANFAMV
ORF2	791-802	439.2332	1314.6777	1314.6754	2	0	KHGPLLANFAMV
ORF2	791-802	439.2333	1314.678	1314.6754	2	0	KHGPLLANFAMV
ORF2	791-802	658.3464	1314.6782	1314.6754	2	0	KHGPLLANFAMV
ORF2	791-802	439.2337	1314.6793	1314.6754	3	0	KHGPLLANFAMV
ORF2	807-819	805.9055	1609.7964	1609.7921	3	2	RDQRQEEHSVGLR
ORF2	808-819	485.2421	1452.7045	1452.7069	-2	1	DQRQEEHSVGLR
ORF2	808-819	727.3607	1452.7069	1452.7069	0	1	DQRQEEHSVGLR
ORF2	808-819	364.4301	1453.6914	1453.6909	0	1	DQRQEEHSVGLR
ORF2	808-820	403.2094	1608.8084	1608.808	0	2	DQRQEEHSVGLRI
ORF2	808-820	537.2774	1608.8104	1608.808	1	2	DQRQEEHSVGLRI
ORF2	811-819	519.2542	1036.4938	1036.4938	0	0	QEEHSVGLR
ORF2	811-819	519.2559	1036.4973	1036.4938	3	0	QEEHSVGLR
ORF2	811-819	352.1808	1053.5207	1053.5203	0	0	QEEHSVGLR
ORF2	811-819	352.1809	1053.521	1053.5203	1	0	QEEHSVGLR
ORF2	811-819	527.7678	1053.521	1053.5203	1	0	QEEHSVGLR
ORF2	811-819	527.7679	1053.5213	1053.5203	1	0	QEEHSVGLR
ORF2	811-819	352.1811	1053.5214	1053.5203	1	0	QEEHSVGLR
ORF2	811-819	527.7681	1053.5217	1053.5203	1	0	QEEHSVGLR
ORF2	811-819	352.1814	1053.5225	1053.5203	2	0	QEEHSVGLR
ORF2	811-819	352.1815	1053.5227	1053.5203	2	0	QEEHSVGLR
ORF2	811-820	398.5385	1192.5938	1192.5949	-1	1	QEEHSVGLRI
ORF2	811-820	398.5388	1192.5946	1192.5949	0	1	QEEHSVGLRI
ORF2	811-820	597.3047	1192.5949	1192.5949	0	1	QEEHSVGLRI
ORF2	811-820	398.539	1192.595	1192.5949	0	1	QEEHSVGLRI
ORF2	811-820	404.2143	1209.6211	1209.6214	0	1	QEEHSVGLRI
ORF2	811-820	404.2149	1209.6228	1209.6214	1	1	QEEHSVGLRI
ORF2	811-820	605.8191	1209.6236	1209.6214	2	1	QEEHSVGLRI
ORF2	820-829	366.9044	1097.6915	1097.6921	-1	1	RISTIVGKPI
ORF2	820-829	366.9051	1097.6936	1097.6921	1	1	RISTIVGKPI
ORF2	821-829	471.8021	941.5897	941.591	-1	0	ISTIVGKPI
ORF2	821-829	471.8028	941.5911	941.591	0	0	ISTIVGKPI
ORF2	821-829	471.8029	941.5912	941.591	0	0	ISTIVGKPI
ORF2	821-829	471.8031	941.5917	941.591	1	0	ISTIVGKPI
ORF2	830-838	512.2998	1022.585	1022.5834	2	0	ILYMGTVVK
ORF2	830-838	512.3002	1022.5859	1022.5834	2	0	ILYMGTVVK
ORF2	830-838	512.3005	1022.5865	1022.5834	3	0	ILYMGTVVK
ORF2	830-838	512.3017	1022.5888	1022.5834	5	0	ILYMGTVVK

ORF2	830-838	520.2962	1038.5779	1038.5784	0	0	ILYMGTVVK
ORF2	830-838	520.2971	1038.5797	1038.5784	1	0	ILYMGTVVK
ORF2	830-838	520.2971	1038.5797	1038.5784	1	0	ILYMGTVVK
ORF2	830-838	520.2976	1038.5807	1038.5784	2	0	ILYMGTVVK
ORF2	846-880	758.1841	3785.8841	3785.8577	7	0	LAHAGTGPQLAAHYEPFFDADSTLQDITSDI PLFG
ORF2	881-907	736.5895	2942.3291	2942.33	0	0	GPTHRPADAGPTHMHANDFTNTNQMPA
ORF2	881-907	589.4739	2942.3332	2942.33	1	0	GPTHRPADAGPTHMHANDFTNTNQMPA
ORF2	881-907	740.5894	2958.3286	2958.325	1	0	GPTHRPADAGPTHMHANDFTNTNQMPA
ORF2	881-907	592.6734	2958.3304	2958.325	2	0	GPTHRPADAGPTHMHANDFTNTNQMPA
ORF2	915-923	465.2801	928.5456	928.5454	0	1	RNSIAALGG
ORF2	924-931	358.7186	715.4227	715.4228	0	0	GIVATAGI
ORF2	924-931	358.7191	715.4236	715.4228	1	0	GIVATAGI

Table S3. Peptide mass fingerprinting analysis of p65.

Putative ORF	Start-End	Observed	Mr(expt)	Mr(calc)	pp m	M iss	Sequence
ORF1	267-281	823.3589	1644.7033	1644.7016	1	0	QTDPAAQPEENSGYR
ORF1	267-281	831.8716	1661.7287	1661.7281	0	0	QTDPAAQPEENSGYR
ORF1	267-281	554.9175	1661.7308	1661.7281	2	0	QTDPAAQPEENSGYR
ORF1	267-281	832.3643	1662.7141	1662.7121	1	0	QTDPAAQPEENSGYR
ORF1	282-290	479.7497	957.4849	957.4879	-3	0	RAPAISNER
ORF1	282-290	479.7516	957.4886	957.4879	1	0	RAPAISNER
ORF1	282-290	479.7521	957.4896	957.4879	2	0	RAPAISNER
ORF1	282-290	479.7526	957.4907	957.4879	3	0	RAPAISNER
ORF1	282-290	480.2417	958.4688	958.4719	-3	0	RAPAISNER
ORF1	291-324	1174.2204	3519.6394	3519.6331	2	0	RGEPVDAPAHTGWLSWEDGSLGGNATYY AGSIVR
ORF1	291-324	1760.8299	3519.6453	3519.6331	3	0	RGEPVDAPAHTGWLSWEDGSLGGNATYY AGSIVR
ORF1	291-324	1174.2231	3519.6474	3519.6331	4	0	RGEPVDAPAHTGWLSWEDGSLGGNATYY AGSIVR
ORF1	291-324	1174.2233	3519.6481	3519.6331	4	0	RGEPVDAPAHTGWLSWEDGSLGGNATYY AGSIVR
ORF1	291-324	880.9196	3519.6491	3519.6331	5	0	RGEPVDAPAHTGWLSWEDGSLGGNATYY AGSIVR
ORF1	291-324	1174.5557	3520.6452	3520.6171	8	0	RGEPVDAPAHTGWLSWEDGSLGGNATYY AGSIVR
ORF1	325-342	740.3483	2218.0229	2218.0192	2	0	RTYEHTWNQHLQANFNTY
ORF1	325-342	555.5132	2218.0239	2218.0192	2	0	RTYEHTWNQHLQANFNTY
ORF1	325-342	555.5133	2218.0242	2218.0192	2	0	RTYEHTWNQHLQANFNTY
ORF1	325-342	740.3487	2218.0243	2218.0192	2	0	RTYEHTWNQHLQANFNTY
ORF1	325-342	740.3494	2218.0265	2218.0192	3	0	RTYEHTWNQHLQANFNTY
ORF1	325-342	555.514	2218.0269	2218.0192	4	0	RTYEHTWNQHLQANFNTY
ORF1	325-342	555.5141	2218.0274	2218.0192	4	0	RTYEHTWNQHLQANFNTY
ORF1	325-342	740.35	2218.0282	2218.0192	4	0	RTYEHTWNQHLQANFNTY
ORF1	325-342	740.3501	2218.0285	2218.0192	4	0	RTYEHTWNQHLQANFNTY
ORF1	325-342	740.3518	2218.0337	2218.0192	7	0	RTYEHTWNQHLQANFNTY
ORF1	325-342	555.7609	2219.0144	2219.0032	5	0	RTYEHTWNQHLQANFNTY
ORF1	325-342	1110.5156	2219.0167	2219.0032	6	0	RTYEHTWNQHLQANFNTY
ORF1	325-342	740.6798	2219.0176	2219.0032	7	0	RTYEHTWNQHLQANFNTY
ORF1	325-342	555.7625	2219.0209	2219.0032	8	0	RTYEHTWNQHLQANFNTY
ORF1	325-354	891.1791	3560.6874	3560.6709	5	1	RTYEHTWNQHLQANFNTKYPPGSTIEQNV R

ORF1	325-354	713.3443	3561.6851	3561.6549	8	1	RTYEHTWNQHLQANFNTKYPPGSTIEQNVR
ORF1	325-354	1781.8505	3561.6864	3561.6549	9	1	RTYEHTWNQHLQANFNTKYPPGSTIEQNVR
ORF1	343-354	680.8464	1359.6782	1359.6783	0	0	YPPGSTIEQNVR
ORF1	343-354	680.8469	1359.6792	1359.6783	1	0	YPPGSTIEQNVR
ORF1	343-354	680.8477	1359.6809	1359.6783	2	0	YPPGSTIEQNVR
ORF1	343-354	680.8479	1359.6812	1359.6783	2	0	YPPGSTIEQNVR
ORF1	343-354	454.2344	1359.6813	1359.6783	2	0	YPPGSTIEQNVR
ORF1	355-383	1610.7858	3219.557	3219.5474	3	0	RMSLLLTPQFTADYFWGYGAPSPEAEITA
ORF1	355-383	805.8967	3219.5577	3219.5474	3	0	RMSLLLTPQFTADYFWGYGAPSPEAEITA
ORF1	355-383	1074.1949	3219.5628	3219.5474	5	0	RMSLLLTPQFTADYFWGYGAPSPEAEITA
ORF1	355-383	1079.8558	3236.5457	3236.5264	6	0	RMSLLLTPQFTADYFWGYGAPSPEAEITA
ORF1	384-400	911.9624	1821.9102	1821.9084	1	0	AGFVDSFTVVQMTHVGT
ORF1	384-400	911.9624	1821.9102	1821.9084	1	0	AGFVDSFTVVQMTHVGT
ORF1	384-400	608.3111	1821.9114	1821.9084	2	0	AGFVDSFTVVQMTHVGT
ORF1	384-400	608.3112	1821.9119	1821.9084	2	0	AGFVDSFTVVQMTHVGT
ORF1	384-400	911.9635	1821.9124	1821.9084	2	0	AGFVDSFTVVQMTHVGT
ORF1	384-400	608.3118	1821.9136	1821.9084	3	0	AGFVDSFTVVQMTHVGT
ORF1	384-400	608.3119	1821.9139	1821.9084	3	0	AGFVDSFTVVQMTHVGT
ORF1	384-400	911.9643	1821.9141	1821.9084	3	0	AGFVDSFTVVQMTHVGT
ORF1	384-400	608.3123	1821.9152	1821.9084	4	0	AGFVDSFTVVQMTHVGT
ORF1	384-400	608.3124	1821.9153	1821.9084	4	0	AGFVDSFTVVQMTHVGT
ORF1	384-400	911.9654	1821.9163	1821.9084	4	0	AGFVDSFTVVQMTHVGT
ORF1	384-400	608.313	1821.9171	1821.9084	5	0	AGFVDSFTVVQMTHVGT
ORF1	384-400	613.6428	1837.9066	1837.9033	2	0	AGFVDSFTVVQMTHVGT
ORF1	384-400	919.9607	1837.9068	1837.9033	2	0	AGFVDSFTVVQMTHVGT
ORF1	384-400	613.6434	1837.9083	1837.9033	3	0	AGFVDSFTVVQMTHVGT
ORF1	384-400	613.6434	1837.9083	1837.9033	3	0	AGFVDSFTVVQMTHVGT
ORF1	384-400	919.9615	1837.9084	1837.9033	3	0	AGFVDSFTVVQMTHVGT
ORF1	384-400	613.6436	1837.9091	1837.9033	3	0	AGFVDSFTVVQMTHVGT
ORF1	384-400	613.6438	1837.9096	1837.9033	3	0	AGFVDSFTVVQMTHVGT
ORF1	384-400	613.6439	1837.9098	1837.9033	3	0	AGFVDSFTVVQMTHVGT
ORF1	384-400	613.6442	1837.9108	1837.9033	4	0	AGFVDSFTVVQMTHVGT
ORF1	384-400	613.6446	1837.9121	1837.9033	5	0	AGFVDSFTVVQMTHVGT
ORF1	384-408	901.5005	2701.4795	2701.4626	6	1	AGFVDSFTVVQMTHVGKTILIPVSE
ORF1	401-408	449.7903	897.566	897.5647	1	0	TILIPVSE
ORF1	401-408	449.7903	897.5661	897.5647	1	0	TILIPVSE
ORF1	401-408	449.7904	897.5662	897.5647	2	0	TILIPVSE
ORF1	401-408	449.7905	897.5664	897.5647	2	0	TILIPVSE
ORF1	401-408	449.7907	897.5669	897.5647	2	0	TILIPVSE
ORF1	401-408	449.7908	897.5671	897.5647	3	0	TILIPVSE
ORF1	409-424	914.9678	1827.921	1827.9155	3	0	ETIGYNVPFGLSVYNR
ORF1	409-424	610.3152	1827.9238	1827.9155	5	0	ETIGYNVPFGLSVYNR
ORF1	425-445	1163.5967	2325.1788	2325.1675	5	0	RMPFNSLASITPTSDLYAQIH
ORF1	425-445	1164.09	2326.1655	2326.1515	6	0	RMPFNSLASITPTSDLYAQIH
ORF1	425-445	776.397	2326.1693	2326.1515	8	0	RMPFNSLASITPTSDLYAQIH
ORF1	425-445	781.3983	2341.1732	2341.1624	5	0	RMPFNSLASITPTSDLYAQIH
ORF1	425-445	1171.5947	2341.1749	2341.1624	5	0	RMPFNSLASITPTSDLYAQIH
ORF1	425-445	1172.0836	2342.1527	2342.1464	3	0	RMPFNSLASITPTSDLYAQIH
ORF1	448-455	517.2571	1032.4997	1032.4989	1	1	KVTDDYHM
ORF1	448-455	517.2579	1032.5013	1032.4989	2	1	KVTDDYHM
ORF1	449-455	453.2091	904.4036	904.4039	0	0	VTDDYHM
ORF1	449-455	453.2092	904.4039	904.4039	0	0	VTDDYHM
ORF1	456-463	460.2518	918.4891	918.4957	-7	0	MILTASQG
ORF1	456-463	460.2553	918.496	918.4957	0	0	MILTASQG
ORF1	456-463	468.253	934.4915	934.4906	1	0	MILTASQG
ORF1	456-463	468.2531	934.4917	934.4906	1	0	MILTASQG

ORF1	456-463	468.2532	934.4919	934.4906	1	0	MILTASQG
ORF1	464-477	516.9179	1547.7319	1547.7337	-1	0	GNNHIALGATMYML
ORF1	464-477	774.8748	1547.735	1547.7337	1	0	GNNHIALGATMYML
ORF1	464-477	516.919	1547.7353	1547.7337	1	0	GNNHIALGATMYML
ORF1	464-477	516.9192	1547.7357	1547.7337	1	0	GNNHIALGATMYML
ORF1	464-477	516.9195	1547.7367	1547.7337	2	0	GNNHIALGATMYML
ORF1	464-477	774.8759	1547.7372	1547.7337	2	0	GNNHIALGATMYML
ORF1	464-477	387.9417	1547.7375	1547.7337	2	0	GNNHIALGATMYML
ORF1	464-477	516.9198	1547.7376	1547.7337	3	0	GNNHIALGATMYML
ORF1	464-477	516.9199	1547.7379	1547.7337	3	0	GNNHIALGATMYML
ORF1	464-477	516.9202	1547.7388	1547.7337	3	0	GNNHIALGATMYML
ORF1	464-477	516.9204	1547.7394	1547.7337	4	0	GNNHIALGATMYML
ORF1	464-477	516.9206	1547.7399	1547.7337	4	0	GNNHIALGATMYML
ORF1	464-477	774.8781	1547.7416	1547.7337	5	0	GNNHIALGATMYML
ORF1	464-477	774.8792	1547.7438	1547.7337	7	0	GNNHIALGATMYML
ORF1	464-477	517.2467	1548.7182	1548.7177	0	0	GNNHIALGATMYML
ORF1	464-477	775.3667	1548.7188	1548.7177	1	0	GNNHIALGATMYML
ORF1	464-477	517.2493	1548.726	1548.7177	5	0	GNNHIALGATMYML
ORF1	464-477	775.3721	1548.7297	1548.7177	8	0	GNNHIALGATMYML
ORF1	464-477	782.8727	1563.7309	1563.7286	1	0	GNNHIALGATMYML
ORF1	464-477	782.8729	1563.7313	1563.7286	2	0	GNNHIALGATMYML
ORF1	464-477	522.2511	1563.7316	1563.7286	2	0	GNNHIALGATMYML
ORF1	464-477	391.9403	1563.732	1563.7286	2	0	GNNHIALGATMYML
ORF1	464-477	783.3592	1564.7038	1564.7126	-6	0	GNNHIALGATMYML
ORF1	464-477	522.5794	1564.7163	1564.7126	2	0	GNNHIALGATMYML
ORF1	464-477	783.3689	1564.7232	1564.7126	7	0	GNNHIALGATMYML
ORF1	464-477	522.5821	1564.7246	1564.7126	8	0	GNNHIALGATMYML
ORF1	464-477	527.5826	1579.726	1579.7235	2	0	GNNHIALGATMYML
ORF1	464-477	527.5827	1579.7263	1579.7235	2	0	GNNHIALGATMYML
ORF1	464-477	527.5829	1579.7269	1579.7235	2	0	GNNHIALGATMYML
ORF1	464-477	790.8719	1579.7292	1579.7235	4	0	GNNHIALGATMYML
ORF1	464-477	791.3623	1580.7101	1580.7075	2	0	GNNHIALGATMYML
ORF1	464-477	527.9112	1580.7118	1580.7075	3	0	GNNHIALGATMYML
ORF1	464-477	791.3659	1580.7173	1580.7075	6	0	GNNHIALGATMYML
ORF1	483-502	1097.0333	2192.0521	2192.0498	1	0	VAQEQGANPFITVESEGFNF
ORF1	483-502	1097.0337	2192.0528	2192.0498	1	0	VAQEQGANPFITVESEGFNF
ORF1	483-502	549.0214	2192.0567	2192.0498	3	0	VAQEQGANPFITVESEGFNF
ORF1	483-502	732.0252	2193.0538	2193.0338	9	0	VAQEQGANPFITVESEGFNF
ORF1	503-519	564.6368	1690.8885	1690.889	0	0	FLGAVGSADSVLVETAR
ORF1	503-519	846.4525	1690.8905	1690.889	1	0	FLGAVGSADSVLVETAR
ORF1	503-519	846.4526	1690.8907	1690.889	1	0	FLGAVGSADSVLVETAR
ORF1	503-519	846.4534	1690.8923	1690.889	2	0	FLGAVGSADSVLVETAR
ORF1	503-519	846.4535	1690.8924	1690.889	2	0	FLGAVGSADSVLVETAR
ORF1	503-519	564.6386	1690.8938	1690.889	3	0	FLGAVGSADSVLVETAR
ORF1	503-519	846.4544	1690.8943	1690.889	3	0	FLGAVGSADSVLVETAR
ORF1	503-519	846.4546	1690.8946	1690.889	3	0	FLGAVGSADSVLVETAR
ORF1	503-519	564.6389	1690.8949	1690.889	3	0	FLGAVGSADSVLVETAR
ORF1	503-519	564.6391	1690.8954	1690.889	4	0	FLGAVGSADSVLVETAR
ORF1	503-519	564.6394	1690.8963	1690.889	4	0	FLGAVGSADSVLVETAR
ORF1	503-519	846.4558	1690.897	1690.889	5	0	FLGAVGSADSVLVETAR
ORF1	503-519	564.6397	1690.8974	1690.889	5	0	FLGAVGSADSVLVETAR
ORF1	503-519	846.4562	1690.8978	1690.889	5	0	FLGAVGSADSVLVETAR
ORF1	520-534	561.2678	1680.7815	1680.7786	2	0	RFAANTPMNMVTMPN
ORF1	520-534	841.8817	1681.7489	1681.7626	-8	0	RFAANTPMNMVTMPN
ORF1	520-534	841.8953	1681.7761	1681.7626	8	0	RFAANTPMNMVTMPN
ORF1	520-534	849.3942	1696.7738	1696.7735	0	0	RFAANTPMNMVTMPN
ORF1	520-534	566.5993	1696.7759	1696.7735	1	0	RFAANTPMNMVTMPN
ORF1	520-534	566.5996	1696.7769	1696.7735	2	0	RFAANTPMNMVTMPN

ORF1	520-534	849.397	1696.7794	1696.7735	3	0	RFAANTPMNMVTMPN
ORF1	520-534	857.3919	1712.7693	1712.7684	0	0	RFAANTPMNMVTMPN
ORF1	520-534	571.9306	1712.77	1712.7684	1	0	RFAANTPMNMVTMPN
ORF1	520-534	857.3927	1712.7708	1712.7684	1	0	RFAANTPMNMVTMPN
ORF1	520-534	857.3931	1712.7716	1712.7684	2	0	RFAANTPMNMVTMPN
ORF1	520-534	571.932	1712.7743	1712.7684	3	0	RFAANTPMNMVTMPN
ORF1	520-534	865.3901	1728.7656	1728.7634	1	0	RFAANTPMNMVTMPN
ORF1	520-534	865.3907	1728.7669	1728.7634	2	0	RFAANTPMNMVTMPN
ORF1	520-534	865.3917	1728.7689	1728.7634	3	0	RFAANTPMNMVTMPN
ORF1	520-534	865.3924	1728.7702	1728.7634	4	0	RFAANTPMNMVTMPN
ORF1	535-552	749.9994	2246.9764	2246.9725	2	0	NAVEDEHLYMYYLCGNTL
ORF1	535-552	750	2246.978	2246.9725	2	0	NAVEDEHLYMYYLCGNTL
ORF1	535-552	750.3259	2247.9559	2247.9565	0	0	NAVEDEHLYMYYLCGNTL
ORF1	535-552	1124.989	2247.9634	2247.9565	3	0	NAVEDEHLYMYYLCGNTL
ORF1	535-552	1124.9906	2247.9666	2247.9565	4	0	NAVEDEHLYMYYLCGNTL
ORF1	535-552	755.3304	2262.9693	2262.9674	1	0	NAVEDEHLYMYYLCGNTL
ORF1	535-552	1132.4951	2262.9757	2262.9674	4	0	NAVEDEHLYMYYLCGNTL
ORF1	535-552	1132.4972	2262.9798	2262.9674	5	0	NAVEDEHLYMYYLCGNTL
ORF1	535-552	755.6598	2263.9575	2263.9514	3	0	NAVEDEHLYMYYLCGNTL
ORF1	553-566	551.303	1650.8872	1650.8842	2	1	LRIPWQQDANSPVD
ORF1	553-566	826.4511	1650.8877	1650.8842	2	1	LRIPWQQDANSPVD
ORF1	553-580	837.1745	3344.6691	3344.6612	2	2	LRIPWQQDANSPVKDLFSCLDAYVPEQF
ORF1	555-566	691.858	1381.7014	1381.699	2	0	IPWQQDANSPVD
ORF1	555-566	692.3508	1382.6871	1382.683	3	0	IPWQQDANSPVD
ORF1	555-580	1026.1694	3075.4864	3075.476	3	1	IPWQQDANSPVKDLFSCLDAYVPEQF
ORF1	555-580	769.879	3075.4868	3075.476	4	1	IPWQQDANSPVKDLFSCLDAYVPEQF
ORF1	555-580	1539.2506	3076.4867	3076.46	9	1	IPWQQDANSPVKDLFSCLDAYVPEQF
ORF1	567-580	856.9027	1711.7909	1711.7876	2	0	DLFSCLDAYVPEQF
ORF1	567-580	856.9034	1711.7922	1711.7876	3	0	DLFSCLDAYVPEQF
ORF1	567-580	856.9051	1711.7957	1711.7876	5	0	DLFSCLDAYVPEQF
ORF1	567-580	856.9054	1711.7963	1711.7876	5	0	DLFSCLDAYVPEQF
ORF1	581-591	644.357	1286.6994	1286.6983	1	0	FILTPIANDQI
ORF1	581-591	644.3577	1286.7008	1286.6983	2	0	FILTPIANDQI
ORF1	581-591	429.9077	1286.7013	1286.6983	2	0	FILTPIANDQI
ORF1	581-591	644.3593	1286.704	1286.6983	4	0	FILTPIANDQI
ORF1	581-591	644.3598	1286.7051	1286.6983	5	0	FILTPIANDQI
ORF1	581-591	644.3598	1286.7051	1286.6983	5	0	FILTPIANDQI
ORF1	581-591	644.8495	1287.6845	1287.6823	2	0	FILTPIANDQI
ORF1	581-601	1183.1493	2364.2841	2364.2801	2	1	FILTPIANDQRIEPLPVGTDI
ORF1	581-601	789.1026	2364.286	2364.2801	2	1	FILTPIANDQRIEPLPVGTDI
ORF1	581-601	592.08	2364.2911	2364.2801	5	1	FILTPIANDQRIEPLPVGTDI
ORF1	581-601	1183.6438	2365.273	2365.2641	4	1	FILTPIANDQRIEPLPVGTDI
ORF1	581-601	789.4316	2365.2731	2365.2641	4	1	FILTPIANDQRIEPLPVGTDI
ORF1	592-601	366.205	1095.5933	1095.5924	1	0	IEPLPVGTDI
ORF1	592-601	548.8044	1095.5943	1095.5924	2	0	IEPLPVGTDI
ORF1	592-601	548.8045	1095.5944	1095.5924	2	0	IEPLPVGTDI
ORF1	604-616	753.9306	1505.8467	1505.8453	1	1	RVDSYLLETIVAY
ORF1	604-616	502.9567	1505.8483	1505.8453	2	1	RVDSYLLETIVAY
ORF1	604-616	753.9324	1505.8503	1505.8453	3	1	RVDSYLLETIVAY
ORF1	605-616	450.9226	1349.7459	1349.7442	1	0	VDSYLLETIVAY
ORF1	605-616	675.8806	1349.7466	1349.7442	2	0	VDSYLLETIVAY
ORF1	605-616	675.8814	1349.7483	1349.7442	3	0	VDSYLLETIVAY
ORF1	605-616	675.8819	1349.7492	1349.7442	4	0	VDSYLLETIVAY
ORF1	617-631	931.9736	1861.9327	1861.9298	2	0	YFTTHNLWGQLPVMA
ORF1	617-631	621.6524	1861.9353	1861.9298	3	0	YFTTHNLWGQLPVMA
ORF1	617-631	621.6527	1861.9363	1861.9298	4	0	YFTTHNLWGQLPVMA
ORF1	617-631	621.6538	1861.9395	1861.9298	5	0	YFTTHNLWGQLPVMA
ORF1	617-631	621.9801	1862.9184	1862.9138	2	0	YFTTHNLWGQLPVMA

ORF1	617-631	621.9831	1862.9275	1862.9138	7	0	YFTTHNLWGQLPVMA
ORF1	617-631	470.4892	1877.9277	1877.9247	2	0	YFTTHNLWGQLPVMA
ORF1	617-631	939.9722	1877.9299	1877.9247	3	0	YFTTHNLWGQLPVMA
ORF1	617-631	626.984	1877.9301	1877.9247	3	0	YFTTHNLWGQLPVMA
ORF1	617-631	626.9848	1877.9327	1877.9247	4	0	YFTTHNLWGQLPVMA
ORF1	617-631	939.9743	1877.934	1877.9247	5	0	YFTTHNLWGQLPVMA
ORF1	632-648	598.6743	1793.0009	1792.9988	1	0	AFAWAILAHPATAVNIF
ORF1	632-648	449.258	1793.0028	1792.9988	2	0	AFAWAILAHPATAVNIF
ORF1	632-648	598.6758	1793.0055	1792.9988	4	0	AFAWAILAHPATAVNIF
ORF1	632-648	598.6765	1793.0077	1792.9988	5	0	AFAWAILAHPATAVNIF
ORF1	632-648	898.0059	1793.9973	1793.9828	8	0	AFAWAILAHPATAVNIF
ORF1	649-670	1256.1253	2510.2361	2510.2376	-1	0	FPAPMHTSELQLNLWPNTTASV
ORF1	649-670	837.753	2510.2371	2510.2376	0	0	FPAPMHTSELQLNLWPNTTASV
ORF1	649-670	628.5673	2510.2399	2510.2376	1	0	FPAPMHTSELQLNLWPNTTASV
ORF1	649-670	838.0833	2511.228	2511.2216	3	0	FPAPMHTSELQLNLWPNTTASV
ORF1	649-670	838.4139	2512.2198	2512.2056	6	0	FPAPMHTSELQLNLWPNTTASV
ORF1	649-670	1264.1248	2526.235	2526.2325	1	0	FPAPMHTSELQLNLWPNTTASV
ORF1	649-670	632.5678	2526.2421	2526.2325	4	0	FPAPMHTSELQLNLWPNTTASV
ORF1	649-670	1264.6206	2527.2267	2527.2165	4	0	FPAPMHTSELQLNLWPNTTASV
ORF1	674-693	722.0676	2163.1809	2163.18	0	1	ILGDKDHFTSLHVATVIAAN
ORF1	674-693	1082.5978	2163.181	2163.18	0	1	ILGDKDHFTSLHVATVIAAN
ORF1	674-693	1082.5993	2163.184	2163.18	2	1	ILGDKDHFTSLHVATVIAAN
ORF1	674-693	722.0688	2163.1845	2163.18	2	1	ILGDKDHFTSLHVATVIAAN
ORF1	674-693	722.0688	2163.1845	2163.18	2	1	ILGDKDHFTSLHVATVIAAN
ORF1	674-693	541.8035	2163.1848	2163.18	2	1	ILGDKDHFTSLHVATVIAAN
ORF1	674-693	541.8036	2163.1853	2163.18	2	1	ILGDKDHFTSLHVATVIAAN
ORF1	674-693	433.6444	2163.1855	2163.18	3	1	ILGDKDHFTSLHVATVIAAN
ORF1	674-693	433.6444	2163.1858	2163.18	3	1	ILGDKDHFTSLHVATVIAAN
ORF1	674-693	541.8038	2163.1861	2163.18	3	1	ILGDKDHFTSLHVATVIAAN
ORF1	674-693	541.8039	2163.1864	2163.18	3	1	ILGDKDHFTSLHVATVIAAN
ORF1	674-693	541.804	2163.1869	2163.18	3	1	ILGDKDHFTSLHVATVIAAN
ORF1	674-693	541.804	2163.187	2163.18	3	1	ILGDKDHFTSLHVATVIAAN
ORF1	674-693	722.0697	2163.1872	2163.18	3	1	ILGDKDHFTSLHVATVIAAN
ORF1	674-693	722.0699	2163.1879	2163.18	4	1	ILGDKDHFTSLHVATVIAAN
ORF1	674-693	541.8043	2163.1879	2163.18	4	1	ILGDKDHFTSLHVATVIAAN
ORF1	674-693	433.6449	2163.1883	2163.18	4	1	ILGDKDHFTSLHVATVIAAN
ORF1	674-693	722.0701	2163.1885	2163.18	4	1	ILGDKDHFTSLHVATVIAAN
ORF1	674-693	722.0704	2163.1894	2163.18	4	1	ILGDKDHFTSLHVATVIAAN
ORF1	674-693	541.8047	2163.1896	2163.18	4	1	ILGDKDHFTSLHVATVIAAN
ORF1	674-693	541.8047	2163.1897	2163.18	4	1	ILGDKDHFTSLHVATVIAAN
ORF1	674-693	722.0706	2163.1898	2163.18	5	1	ILGDKDHFTSLHVATVIAAN
ORF1	674-693	433.6452	2163.1899	2163.18	5	1	ILGDKDHFTSLHVATVIAAN
ORF1	674-693	433.6455	2163.191	2163.18	5	1	ILGDKDHFTSLHVATVIAAN
ORF1	679-693	410.2257	1636.8736	1636.8685	3	0	DHFTSLHVATVIAAN
ORF1	679-693	819.4446	1636.8747	1636.8685	4	0	DHFTSLHVATVIAAN
ORF1	694-720	974.1541	2919.4405	2919.4324	3	0	NCEEVLTDAIVSATTAVGIPYTHPAYA
ORF1	694-720	584.8965	2919.4463	2919.4324	5	0	NCEEVLTDAIVSATTAVGIPYTHPAYA
ORF1	694-720	1460.7328	2919.451	2919.4324	6	0	NCEEVLTDAIVSATTAVGIPYTHPAYA
ORF1	694-720	731.1134	2920.4245	2920.4164	3	0	NCEEVLTDAIVSATTAVGIPYTHPAYA
ORF1	694-720	974.4824	2920.4253	2920.4164	3	0	NCEEVLTDAIVSATTAVGIPYTHPAYA
ORF1	694-720	974.4836	2920.4289	2920.4164	4	0	NCEEVLTDAIVSATTAVGIPYTHPAYA
ORF1	694-720	1461.2229	2920.4312	2920.4164	5	0	NCEEVLTDAIVSATTAVGIPYTHPAYA
ORF1	721-729	501.7651	1001.5156	1001.5142	1	0	ATVEQELGH
ORF1	721-729	501.7652	1001.5159	1001.5142	2	0	ATVEQELGH
ORF1	773-780	490.2823	978.55	978.5498	0	1	RLDTKLFA
ORF1	781-804	904.4299	2710.2678	2710.2762	-3	0	ASSYLVVNEEPEGEGWQLVFSENR
ORF1	781-804	1356.148	2710.2814	2710.2762	2	0	ASSYLVVNEEPEGEGWQLVFSENR
ORF1	781-804	904.435	2710.283	2710.2762	3	0	ASSYLVVNEEPEGEGWQLVFSENR

ORF1	781-804	1356.1501	2710.2857	2710.2762	4	0	ASSYLVVNEEPEGEGWQLVFSENR
ORF1	781-804	904.4362	2710.2868	2710.2762	4	0	ASSYLVVNEEPEGEGWQLVFSENR
ORF1	781-804	1356.1516	2710.2886	2710.2762	5	0	ASSYLVVNEEPEGEGWQLVFSENR
ORF1	781-804	904.4371	2710.2894	2710.2762	5	0	ASSYLVVNEEPEGEGWQLVFSENR
ORF1	781-805	1434.1985	2866.3825	2866.3773	2	1	ASSYLVVNEEPEGEGWQLVFSENKK
ORF1	781-805	717.6038	2866.3859	2866.3773	3	1	ASSYLVVNEEPEGEGWQLVFSENKK
ORF1	781-805	956.4694	2866.3865	2866.3773	3	1	ASSYLVVNEEPEGEGWQLVFSENKK
ORF1	781-805	956.8033	2867.3882	2867.3613	9	1	ASSYLVVNEEPEGEGWQLVFSENKK
ORF1	816-828	501.9455	1502.8148	1502.8093	4	1	SREALVATWLTES
ORF1	816-828	752.4156	1502.8166	1502.8093	5	1	SREALVATWLTES
ORF1	818-828	621.8434	1241.6722	1241.6656	5	0	EALVATWLTES
ORF1	818-828	420.8993	1259.6762	1259.6761	0	0	EALVATWLTES
ORF1	818-828	630.8468	1259.679	1259.6761	2	0	EALVATWLTES
ORF1	818-828	630.8468	1259.6791	1259.6761	2	0	EALVATWLTES
ORF1	818-828	630.8471	1259.6797	1259.6761	3	0	EALVATWLTES
ORF1	818-828	630.8473	1259.68	1259.6761	3	0	EALVATWLTES
ORF1	818-828	630.8473	1259.6801	1259.6761	3	0	EALVATWLTES
ORF1	818-828	630.8486	1259.6827	1259.6761	5	0	EALVATWLTES
ORF1	834-862	1046.8492	3137.5259	3137.5227	1	0	ASDITYYQHETAEVDSTVVLPVMLGNTGN
ORF1	834-862	785.3896	3137.5292	3137.5227	2	0	ASDITYYQHETAEVDSTVVLPVMLGNTGN
ORF1	834-862	1046.8514	3137.5324	3137.5227	3	0	ASDITYYQHETAEVDSTVVLPVMLGNTGN
ORF1	834-862	1046.8531	3137.5376	3137.5227	5	0	ASDITYYQHETAEVDSTVVLPVMLGNTGN
ORF1	834-862	785.3918	3137.538	3137.5227	5	0	ASDITYYQHETAEVDSTVVLPVMLGNTGN
ORF1	834-862	1046.8541	3137.5403	3137.5227	6	0	ASDITYYQHETAEVDSTVVLPVMLGNTGN
ORF1	834-862	1046.8555	3137.5447	3137.5227	7	0	ASDITYYQHETAEVDSTVVLPVMLGNTGN
ORF1	834-862	1570.2642	3138.5138	3138.5067	2	0	ASDITYYQHETAEVDSTVVLPVMLGNTGN
ORF1	834-862	1047.1831	3138.5275	3138.5067	7	0	ASDITYYQHETAEVDSTVVLPVMLGNTGN
ORF1	834-862	1570.2729	3138.5313	3138.5067	8	0	ASDITYYQHETAEVDSTVVLPVMLGNTGN
ORF1	834-862	1570.7659	3139.5172	3139.4907	8	0	ASDITYYQHETAEVDSTVVLPVMLGNTGN
ORF1	834-862	1052.1811	3153.5213	3153.5176	1	0	ASDITYYQHETAEVDSTVVLPVMLGNTGN
ORF1	834-862	1052.1816	3153.523	3153.5176	2	0	ASDITYYQHETAEVDSTVVLPVMLGNTGN
ORF1	834-862	1577.7704	3153.5262	3153.5176	3	0	ASDITYYQHETAEVDSTVVLPVMLGNTGN
ORF1	834-862	1052.1829	3153.5269	3153.5176	3	0	ASDITYYQHETAEVDSTVVLPVMLGNTGN
ORF1	834-862	789.3905	3153.533	3153.5176	5	0	ASDITYYQHETAEVDSTVVLPVMLGNTGN
ORF1	834-862	1052.5168	3154.5287	3154.5016	9	0	ASDITYYQHETAEVDSTVVLPVMLGNTGN
ORF1	834-862	789.6396	3154.5295	3154.5016	9	0	ASDITYYQHETAEVDSTVVLPVMLGNTGN
ORF1	834-862	789.6401	3154.5314	3154.5016	9	0	ASDITYYQHETAEVDSTVVLPVMLGNTGN
ORF1	865-880	650.3515	1948.0328	1948.0319	0	0	LTNLAGRPQLWDYFVG
ORF1	865-880	488.0155	1948.0329	1948.0319	1	0	LTNLAGRPQLWDYFVG
ORF1	865-880	650.3524	1948.0355	1948.0319	2	0	LTNLAGRPQLWDYFVG
ORF1	865-880	975.0251	1948.0357	1948.0319	2	0	LTNLAGRPQLWDYFVG
ORF1	865-880	650.3528	1948.0364	1948.0319	2	0	LTNLAGRPQLWDYFVG
ORF1	865-880	650.3538	1948.0395	1948.0319	4	0	LTNLAGRPQLWDYFVG
ORF1	865-880	975.0279	1948.0412	1948.0319	5	0	LTNLAGRPQLWDYFVG
ORF1	881-888	365.7268	729.4391	729.4385	1	0	GLTGLAAS
ORF1	881-888	365.727	729.4395	729.4385	1	0	GLTGLAAS
ORF1	881-888	365.7273	729.4401	729.4385	2	0	GLTGLAAS
ORF1	889-897	449.2558	896.4971	896.4967	0	0	SVVPPAAEN
ORF1	1280-1292	752.405	1502.7955	1502.7902	4	0	RLTIPELETEIMT
ORF1	1343-1351	527.3126	1052.6106	1052.6091	1	0	ELNVAVRPA
ORF1	1425-1436	701.8656	1401.7167	1401.7099	5	0	DNSVLLEESLQR
ORF1	1595-1601	425.7308	849.447	849.4457	2	1	GRGEYLL
ORF1	1690-1701	601.8271	1201.6396	1201.6376	2	0	TSGALGLMPIDK
ORF1	1707-1718	622.319	1242.6235	1242.6204	2	0	DSLAGQPEAVTR
ORF1	1719-1732	737.4063	1472.7981	1472.7908	5	0	RQTPAETLAALLMS
ORF1	1782-1791	556.8023	1111.5901	1111.5873	2	0	RTAHEELLAV
ORF1	1866-1874	490.3001	978.5856	978.5862	-1	0	LDVPLPAVR
ORF1	1917-1922	358.209	714.4034	714.4024	1	0	QTTIPW

	202-209	495.2396	988.4646	988.4648	0	1	QMPTKTEA
ORF2	202-209	495.2401	988.4656	988.4648	1	1	QMPTKTEA
ORF2	202-209	495.76	989.5054	989.4964	9	1	QMPTKTEA
ORF2	214-224	361.1686	1080.4839	1080.4836	0	0	AAGVYGDDSAR
ORF2	214-224	541.2497	1080.4848	1080.4836	1	0	AAGVYGDDSAR
ORF2	214-224	541.2498	1080.4851	1080.4836	1	0	AAGVYGDDSAR
ORF2	214-224	541.2504	1080.4863	1080.4836	2	0	AAGVYGDDSAR
ORF2	225-234	378.2085	1131.6038	1131.6023	1	0	RSIVEEVVER
ORF2	225-234	566.8095	1131.6044	1131.6023	2	0	RSIVEEVVER
ORF2	225-235	430.242	1287.7042	1287.7034	1	1	RSIVEEVVEKG
ORF2	225-235	644.8594	1287.7043	1287.7034	1	1	RSIVEEVVEKG
ORF2	225-235	430.2432	1287.7079	1287.7034	3	1	RSIVEEVVEKG
ORF2	225-235	644.8616	1287.7086	1287.7034	4	1	RSIVEEVVEKG
ORF2	235-258	1381.1898	2760.3651	2760.362	1	1	RGLWESIVTWTPDEQLANAQAYGV
ORF2	235-258	921.4631	2761.3676	2761.346	8	1	RGLWESIVTWTPDEQLANAQAYGV
ORF2	236-258	1303.146	2604.2774	2604.2608	6	0	GLWESIVTWTPDEQLANAQAYGV
ORF2	259-277	1006.0675	2010.1204	2010.1184	1	0	VLVQPTVHGMLSLTSTISA
ORF2	259-277	671.0475	2010.1208	2010.1184	1	0	VLVQPTVHGMLSLTSTISA
ORF2	259-277	671.0482	2010.1227	2010.1184	2	0	VLVQPTVHGMLSLTSTISA
ORF2	259-277	1006.0702	2010.1259	2010.1184	4	0	VLVQPTVHGMLSLTSTISA
ORF2	259-277	671.0493	2010.1261	2010.1184	4	0	VLVQPTVHGMLSLTSTISA
ORF2	259-277	671.05	2010.1283	2010.1184	5	0	VLVQPTVHGMLSLTSTISA
ORF2	259-277	676.3793	2026.116	2026.1133	1	0	VLVQPTVHGMLSLTSTISA
ORF2	259-277	1014.0655	2026.1164	2026.1133	2	0	VLVQPTVHGMLSLTSTISA
ORF2	259-277	676.3795	2026.1168	2026.1133	2	0	VLVQPTVHGMLSLTSTISA
ORF2	259-277	676.38	2026.1181	2026.1133	2	0	VLVQPTVHGMLSLTSTISA
ORF2	259-277	676.3809	2026.1209	2026.1133	4	0	VLVQPTVHGMLSLTSTISA
ORF2	259-277	676.3812	2026.1218	2026.1133	4	0	VLVQPTVHGMLSLTSTISA
ORF2	259-277	676.3819	2026.1239	2026.1133	5	0	VLVQPTVHGMLSLTSTISA
ORF2	278-285	432.2533	862.492	862.4912	1	0	ALGTINFD
ORF2	278-285	432.2535	862.4925	862.4912	1	0	ALGTINFD
ORF2	278-285	432.7451	863.4757	863.4752	0	0	ALGTINFD
ORF2	278-296	668.7104	2003.1095	2003.1051	2	1	ALGTINFKDTLSVLAPSTI
ORF2	278-296	1002.5635	2003.1125	2003.1051	4	1	ALGTINFKDTLSVLAPSTI
ORF2	278-296	669.0429	2004.1069	2004.0891	9	1	ALGTINFKDTLSVLAPSTI
ORF2	286-296	580.3202	1158.6259	1158.6245	1	0	DTLSVLAPSTI
ORF2	286-296	387.2166	1158.628	1158.6245	3	0	DTLSVLAPSTI
ORF2	300-310	697.8492	1393.6839	1393.6812	2	0	IAICWGYNLEL
ORF2	300-310	697.8502	1393.6858	1393.6812	3	0	IAICWGYNLEL
ORF2	300-310	697.8503	1393.686	1393.6812	3	0	IAICWGYNLEL
ORF2	300-310	697.8506	1393.6867	1393.6812	4	0	IAICWGYNLEL
ORF2	300-310	697.8516	1393.6886	1393.6812	5	0	IAICWGYNLEL
ORF2	316-338	850.4053	2548.194	2548.187	3	0	VGTAYSDEYGVALWGTRPNYSDA
ORF2	316-338	850.7321	2549.1746	2549.171	1	0	VGTAYSDEYGVALWGTRPNYSDA
ORF2	316-338	638.3052	2549.1916	2549.171	8	0	VGTAYSDEYGVALWGTRPNYSDA
ORF2	339-347	352.5504	1054.6293	1054.6287	1	0	ARPYKPPVT
ORF2	339-347	352.5505	1054.6296	1054.6287	1	0	ARPYKPPVT
ORF2	339-347	352.5505	1054.6298	1054.6287	1	0	ARPYKPPVT
ORF2	339-347	352.5509	1054.6309	1054.6287	2	0	ARPYKPPVT
ORF2	339-347	352.5509	1054.631	1054.6287	2	0	ARPYKPPVT
ORF2	339-347	528.3228	1054.631	1054.6287	2	0	ARPYKPPVT
ORF2	339-347	528.3231	1054.6317	1054.6287	3	0	ARPYKPPVT
ORF2	339-347	352.5519	1054.6337	1054.6287	5	0	ARPYKPPVT
ORF2	339-347	352.5519	1054.6339	1054.6287	5	0	ARPYKPPVT
ORF2	348-359	452.5698	1354.6876	1354.6881	0	0	TIQTGVGYYTPV
ORF2	348-359	678.3521	1354.6896	1354.6881	1	0	TIQTGVGYYTPV
ORF2	348-359	678.3526	1354.6906	1354.6881	2	0	TIQTGVGYYTPV
ORF2	348-359	678.353	1354.6914	1354.6881	2	0	TIQTGVGYYTPV

ORF2	348-359	678.3552	1354.6958	1354.6881	6	0	TIQTGVGYYTPV
ORF2	360-375	816.8982	1631.7818	1631.7825	0	0	VMANEIVAGSVDNADL
ORF2	360-375	816.8987	1631.7829	1631.7825	0	0	VMANEIVAGSVDNADL
ORF2	360-375	544.9366	1631.788	1631.7825	3	0	VMANEIVAGSVDNADL
ORF2	360-375	817.3914	1632.7682	1632.7665	1	0	VMANEIVAGSVDNADL
ORF2	360-375	824.8972	1647.7799	1647.7774	2	0	VMANEIVAGSVDNADL
ORF2	360-375	550.2674	1647.7803	1647.7774	2	0	VMANEIVAGSVDNADL
ORF2	360-375	825.3916	1648.7686	1648.7614	4	0	VMANEIVAGSVDNADL
ORF2	360-380	1101.0662	2200.1178	2200.1158	1	1	VMANEIVAGSVDNADKLVEAD
ORF2	360-380	734.3806	2200.12	2200.1158	2	1	VMANEIVAGSVDNADKLVEAD
ORF2	360-380	734.3816	2200.1231	2200.1158	3	1	VMANEIVAGSVDNADKLVEAD
ORF2	360-380	551.0383	2200.1241	2200.1158	4	1	VMANEIVAGSVDNADKLVEAD
ORF2	360-380	1101.5585	2201.1025	2201.0998	1	1	VMANEIVAGSVDNADKLVEAD
ORF2	360-380	734.7089	2201.1047	2201.0998	2	1	VMANEIVAGSVDNADKLVEAD
ORF2	360-380	739.7107	2216.1102	2216.1107	0	1	VMANEIVAGSVDNADKLVEAD
ORF2	360-380	1109.064	2216.1134	2216.1107	1	1	VMANEIVAGSVDNADKLVEAD
ORF2	360-380	739.7123	2216.1151	2216.1107	2	1	VMANEIVAGSVDNADKLVEAD
ORF2	360-380	739.7131	2216.1174	2216.1107	3	1	VMANEIVAGSVDNADKLVEAD
ORF2	360-380	555.0367	2216.1179	2216.1107	3	1	VMANEIVAGSVDNADKLVEAD
ORF2	360-380	1109.5548	2217.0951	2217.0947	0	1	VMANEIVAGSVDNADKLVEAD
ORF2	381-388	517.2205	1032.4264	1032.4222	4	0	DMTNYFDM
ORF2	381-388	517.7108	1033.4071	1033.4063	1	0	DMTNYFDM
ORF2	381-388	525.2164	1048.4183	1048.4172	1	0	DMTNYFDM
ORF2	381-388	525.2165	1048.4184	1048.4172	1	0	DMTNYFDM
ORF2	381-388	525.2168	1048.419	1048.4172	2	0	DMTNYFDM
ORF2	381-388	525.2177	1048.4208	1048.4172	3	0	DMTNYFDM
ORF2	381-390	431.5273	1291.56	1291.5577	2	1	DMTNYFDKMK
ORF2	381-390	646.7874	1291.5601	1291.5577	2	1	DMTNYFDKMK
ORF2	381-390	654.7834	1307.5523	1307.5526	0	1	DMTNYFDKMK
ORF2	381-390	436.859	1307.5551	1307.5526	2	1	DMTNYFDKMK
ORF2	381-390	662.7813	1323.5481	1323.5475	0	1	DMTNYFDKMK
ORF2	381-390	442.19	1323.5481	1323.5475	0	1	DMTNYFDKMK
ORF2	391-398	459.2949	916.5752	916.5746	1	1	KIPAIFTA
ORF2	391-398	459.2953	916.5761	916.5746	2	1	KIPAIFTA
ORF2	391-398	459.2955	916.5764	916.5746	2	1	KIPAIFTA
ORF2	391-403	463.2825	1386.8258	1386.8235	2	2	KIPAIFTKAGDVS
ORF2	392-398	395.2478	788.481	788.4796	2	0	IPAIFTA
ORF2	392-398	395.248	788.4815	788.4796	2	0	IPAIFTA
ORF2	392-398	395.2482	788.4818	788.4796	3	0	IPAIFTA
ORF2	404-428	954.8124	2861.4153	2861.3993	6	0	SQYGDNHVGMLVQWWLMLAAVTQAE
ORF2	404-428	716.3621	2861.4191	2861.3993	7	0	SQYGDNHVGMLVQWWLMLAAVTQAE
ORF2	404-453	1888.2117	5661.6132	5661.6153	0	1	SQYGDNHVGMLVQWWLMLAAVTQAKEQ GAACTTQLYQEALDDYTLDVTPA
ORF2	429-453	1415.6549	2829.2953	2829.3015	-2	0	EQGAACTTQLYQEALDDYTLDVTPA
ORF2	429-453	1415.6582	2829.3019	2829.3015	0	0	EQGAACTTQLYQEALDDYTLDVTPA
ORF2	429-453	1415.6607	2829.3068	2829.3015	2	0	EQGAACTTQLYQEALDDYTLDVTPA
ORF2	429-453	944.1103	2829.3092	2829.3015	3	0	EQGAACTTQLYQEALDDYTLDVTPA
ORF2	429-453	708.584	2830.3068	2830.2855	8	0	EQGAACTTQLYQEALDDYTLDVTPA
ORF2	454-467	422.2058	1684.7939	1684.7917	1	1	ADSRETTEWHINAL
ORF2	458-467	413.5356	1237.585	1237.584	1	0	ETTEWHINAL
ORF2	458-467	619.8008	1237.5871	1237.584	3	0	ETTEWHINAL
ORF2	458-467	620.2912	1238.5678	1238.568	0	0	ETTEWHINAL
ORF2	458-467	419.5384	1255.5934	1255.5945	-1	0	ETTEWHINAL
ORF2	458-467	628.8054	1255.5963	1255.5945	1	0	ETTEWHINAL
ORF2	458-467	419.5396	1255.5968	1255.5945	2	0	ETTEWHINAL
ORF2	458-467	628.806	1255.5975	1255.5945	2	0	ETTEWHINAL
ORF2	458-467	419.5401	1255.5983	1255.5945	3	0	ETTEWHINAL
ORF2	458-467	419.5401	1255.5984	1255.5945	3	0	ETTEWHINAL

ORF2	458-467	628.8069	1255.5993	1255.5945	4	0	ETTEWHINAL
ORF2	458-467	419.5405	1255.5998	1255.5945	4	0	ETTEWHINAL
ORF2	468-477	580.8009	1159.5872	1159.5873	0	0	LAEVWSQAEI
ORF2	468-477	387.5366	1159.5879	1159.5873	0	0	LAEVWSQAEI
ORF2	478-507	832.197	3324.7587	3324.7437	5	0	IKPFNMVLPSDSATHVDVVHLMFLSGMLVN
ORF2	478-507	1114.5956	3340.7649	3340.7386	8	0	IKPFNMVLPSDSATHVDVVHLMFLSGMLVN
ORF2	478-507	836.4432	3341.7438	3341.7226	6	0	IKPFNMVLPSDSATHVDVVHLMFLSGMLVN
ORF2	478-507	836.4445	3341.7487	3341.7226	8	0	IKPFNMVLPSDSATHVDVVHLMFLSGMLVN
ORF2	478-507	672.3564	3356.7457	3356.7335	4	0	IKPFNMVLPSDSATHVDVVHLMFLSGMLVN
ORF2	478-507	840.1942	3356.7477	3356.7335	4	0	IKPFNMVLPSDSATHVDVVHLMFLSGMLVN
ORF2	478-507	675.5545	3372.7361	3372.7284	2	0	IKPFNMVLPSDSATHVDVVHLMFLSGMLVN
ORF2	508-514	457.2425	912.4704	912.4705	0	1	NDKFVYG
ORF2	508-514	457.2432	912.4719	912.4705	1	1	NDKFVYG
ORF2	508-514	457.7345	913.4545	913.4545	0	1	NDKFVYG
ORF2	515-532	705.7099	2114.1079	2114.1048	1	1	GRNLGILPTYYLYQTDVD
ORF2	515-536	666.3547	2661.3898	2661.3802	4	2	GRNLGILPTYYLYQTDVKDYLI
ORF2	517-532	951.0075	1900.0004	1899.9982	1	0	NLGILPTYYLYQTDVD
ORF2	517-532	634.3411	1900.0014	1899.9982	2	0	NLGILPTYYLYQTDVD
ORF2	517-532	951.5034	1900.9923	1900.9822	5	0	NLGILPTYYLYQTDVD
ORF2	517-532	951.5046	1900.9946	1900.9822	7	0	NLGILPTYYLYQTDVD
ORF2	517-536	1224.6464	2447.2783	2447.2736	2	1	NLGILPTYYLYQTDVKDYLI
ORF2	517-536	816.7686	2447.2839	2447.2736	4	1	NLGILPTYYLYQTDVKDYLI
ORF2	517-536	816.7695	2447.2867	2447.2736	5	1	NLGILPTYYLYQTDVKDYLI
ORF2	517-536	817.0957	2448.2654	2448.2576	3	1	NLGILPTYYLYQTDVKDYLI
ORF2	517-536	817.0968	2448.2684	2448.2576	4	1	NLGILPTYYLYQTDVKDYLI
ORF2	517-536	1225.147	2448.2794	2448.2576	9	1	NLGILPTYYLYQTDVKDYLI
ORF2	537-549	757.9159	1513.8172	1513.8174	0	0	IPLCNSLETINLE
ORF2	537-549	505.6133	1513.8181	1513.8174	0	0	IPLCNSLETINLE
ORF2	537-549	758.4102	1514.8059	1514.8014	3	0	IPLCNSLETINLE
ORF2	537-572	798.4302	3987.1148	3987.1027	3	1	IPLCNSLETINLKEQLANTFVVTPGGLEATIVDYVR
ORF2	537-572	1330.0482	3987.1227	3987.1027	5	1	IPLCNSLETINLKEQLANTFVVTPGGLEATIVDYVR
ORF2	550-573	663.108	2648.4028	2648.381	8	1	EQLANTFVVTPGGLEATIVDYVRM
ORF2	550-621	1580.7526	7898.7264	7898.657	9	2	EQLANTFVVTPGGLEATIVDYVRRMGLQDQMTTAQMIGASIMAVASNDGEGHSTNVVPMPNHVQEYDLWISR
ORF2	573-621	1078.1142	5385.5347	5385.5097	5	1	RMGLQDQMTTAQMIGASIMAVASNDGEGHSTNVVPMPNHVQEYDLWISR
ORF2	573-621	1802.8207	5405.4402	5405.4407	0	1	RMGLQDQMTTAQMIGASIMAVASNDGEGHSTNVVPMPNHVQEYDLWISR
ORF2	573-621	1352.3698	5405.4499	5405.4407	2	1	RMGLQDQMTTAQMIGASIMAVASNDGEGHSTNVVPMPNHVQEYDLWISR
ORF2	573-621	1355.885	5419.5109	5419.4676	8	1	RMGLQDQMTTAQMIGASIMAVASNDGEGHSTNVVPMPNHVQEYDLWISR
ORF2	573-621	1359.8821	5435.4992	5435.4625	7	1	RMGLQDQMTTAQMIGASIMAVASNDGEGHSTNVVPMPNHVQEYDLWISR
ORF2	574-621	1046.8901	5229.4141	5229.4086	1	0	MGLQDQMTTAQMIGASIMAVASNDGEGHSTNVVPMPNHVQEYDLWISR
ORF2	574-621	1308.6135	5230.4249	5230.3926	6	0	MGLQDQMTTAQMIGASIMAVASNDGEGHSTNVVPMPNHVQEYDLWISR

ORF2	574-621	1309.3557	5233.3937	5233.3447	9	0	MGLQDQMTTAQMIGASIMAVASNDGEGHS TNVVPMPNHVQEYDLWISR
ORF2	574-621	1749.4825	5245.4257	5245.4035	4	0	MGLQDQMTTAQMIGASIMAVASNDGEGHS TNVVPMPNHVQEYDLWISR
ORF2	574-621	1050.0946	5245.4366	5245.4035	6	0	MGLQDQMTTAQMIGASIMAVASNDGEGHS TNVVPMPNHVQEYDLWISR
ORF2	574-621	1320.8583	5279.404	5279.3614	8	0	MGLQDQMTTAQMIGASIMAVASNDGEGHS TNVVPMPNHVQEYDLWISR
ORF2	574-621	1320.8594	5279.4084	5279.3614	9	0	MGLQDQMTTAQMIGASIMAVASNDGEGHS TNVVPMPNHVQEYDLWISR
ORF2	574-621	1760.8107	5279.4102	5279.3614	9	0	MGLQDQMTTAQMIGASIMAVASNDGEGHS TNVVPMPNHVQEYDLWISR
ORF2	622-659	1374.3949	4120.1629	4120.1303	8	1	RALGVQPSWSLVQGENFTFLSALLQTQGD VLMDTVATL
ORF2	660-667	465.7398	929.4651	929.464	1	0	LMEHVSSD
ORF2	660-667	465.7404	929.4663	929.464	2	0	LMEHVSSD
ORF2	660-667	465.7409	929.4673	929.464	4	0	LMEHVSSD
ORF2	660-667	473.7374	945.4602	945.459	1	0	LMEHVSSD
ORF2	660-667	473.7379	945.4612	945.459	2	0	LMEHVSSD
ORF2	668-690	620.0562	2476.1958	2476.1904	2	0	DLALTTNAGIEEAHAFMSETISG
ORF2	668-690	826.4062	2476.1967	2476.1904	3	0	DLALTTNAGIEEAHAFMSETISG
ORF2	668-690	826.4065	2476.1977	2476.1904	3	0	DLALTTNAGIEEAHAFMSETISG
ORF2	668-690	1239.1067	2476.1988	2476.1904	3	0	DLALTTNAGIEEAHAFMSETISG
ORF2	668-690	826.4086	2476.2039	2476.1904	5	0	DLALTTNAGIEEAHAFMSETISG
ORF2	668-690	1239.597	2477.1794	2477.1744	2	0	DLALTTNAGIEEAHAFMSETISG
ORF2	668-690	826.7338	2477.1795	2477.1744	2	0	DLALTTNAGIEEAHAFMSETISG
ORF2	668-690	1239.598	2477.1815	2477.1744	3	0	DLALTTNAGIEEAHAFMSETISG
ORF2	668-690	1239.6044	2477.1942	2477.1744	8	0	DLALTTNAGIEEAHAFMSETISG
ORF2	668-690	826.7393	2477.1959	2477.1744	9	0	DLALTTNAGIEEAHAFMSETISG
ORF2	668-690	831.7361	2492.1864	2492.1853	0	0	DLALTTNAGIEEAHAFMSETISG
ORF2	668-690	624.0553	2492.1922	2492.1853	3	0	DLALTTNAGIEEAHAFMSETISG
ORF2	668-690	831.7382	2492.1926	2492.1853	3	0	DLALTTNAGIEEAHAFMSETISG
ORF2	668-690	831.739	2492.1952	2492.1853	4	0	DLALTTNAGIEEAHAFMSETISG
ORF2	668-690	831.7393	2492.1959	2492.1853	4	0	DLALTTNAGIEEAHAFMSETISG
ORF2	668-690	831.7409	2492.2009	2492.1853	6	0	DLALTTNAGIEEAHAFMSETISG
ORF2	691-707	640.9879	1919.9419	1919.9418	0	0	GQSQLISAWATYTFGYS
ORF2	691-707	960.9819	1919.9492	1919.9418	4	0	GQSQLISAWATYTFGYS
ORF2	691-707	961.4745	1920.9345	1920.9258	5	0	GQSQLISAWATYTFGYS
ORF2	708-727	726.0754	2175.2043	2175.2011	1	0	SVELQQALNLDASHLVGLID
ORF2	708-727	1088.6095	2175.2044	2175.2011	1	0	SVELQQALNLDASHLVGLID
ORF2	708-727	544.8086	2175.2055	2175.2011	2	0	SVELQQALNLDASHLVGLID
ORF2	708-727	726.4058	2176.1955	2176.1851	5	0	SVELQQALNLDASHLVGLID
ORF2	708-727	726.4089	2176.2048	2176.1851	9	0	SVELQQALNLDASHLVGLID
ORF2	728-761	1806.463	3610.9115	3610.9029	2	0	DTLATGVLRPASLLIANTTIAQGPISMVWDE TTN
ORF2	728-761	723.1903	3610.9153	3610.9029	3	0	DTLATGVLRPASLLIANTTIAQGPISMVWDE TTN
ORF2	728-761	903.9841	3611.9072	3611.8869	6	0	DTLATGVLRPASLLIANTTIAQGPISMVWDE TTN
ORF2	728-761	1205.3091	3612.9054	3612.8709	10	0	DTLATGVLRPASLLIANTTIAQGPISMVWDE TTN
ORF2	728-761	907.7346	3626.9093	3626.8978	3	0	DTLATGVLRPASLLIANTTIAQGPISMVWDE TTN
ORF2	728-761	1209.9773	3626.91	3626.8978	3	0	DTLATGVLRPASLLIANTTIAQGPISMVWDE TTN
ORF2	728-761	1210.3059	3627.8959	3627.8818	4	0	DTLATGVLRPASLLIANTTIAQGPISMVWDE TTN
ORF2	764-776	696.3812	1390.7478	1390.7456	2	1	EAAKIYATNPVSM

ORF2	764-776	464.5901	1390.7484	1390.7456	2	1	EAAKIYATNPVSM
ORF2	768-776	496.7744	991.5343	991.5338	1	0	IYATNPVSM
ORF2	768-776	496.7748	991.5351	991.5338	1	0	IYATNPVSM
ORF2	768-776	496.775	991.5354	991.5338	2	0	IYATNPVSM
ORF2	768-776	496.7751	991.5357	991.5338	2	0	IYATNPVSM
ORF2	768-776	496.7752	991.5359	991.5338	2	0	IYATNPVSM
ORF2	777-790	873.9469	1745.8793	1745.8698	5	0	MLTYIYSNEPQIFT
ORF2	777-790	874.44	1746.8655	1746.8538	7	0	MLTYIYSNEPQIFT
ORF2	777-790	881.9423	1761.8701	1761.8647	3	0	MLTYIYSNEPQIFT
ORF2	777-790	588.2977	1761.8713	1761.8647	4	0	MLTYIYSNEPQIFT
ORF2	791-802	650.3484	1298.6823	1298.6805	1	0	THGPLLANFAMV
ORF2	791-802	433.9017	1298.6831	1298.6805	2	0	THGPLLANFAMV
ORF2	791-802	650.3501	1298.6856	1298.6805	4	0	THGPLLANFAMV
ORF2	791-802	434.2291	1299.6654	1299.6645	1	0	THGPLLANFAMV
ORF2	791-802	658.3448	1314.6751	1314.6754	0	0	THGPLLANFAMV
ORF2	791-802	439.2329	1314.677	1314.6754	1	0	THGPLLANFAMV
ORF2	791-802	658.3459	1314.6773	1314.6754	1	0	THGPLLANFAMV
ORF2	791-802	658.3467	1314.6789	1314.6754	3	0	THGPLLANFAMV
ORF2	791-802	439.2336	1314.679	1314.6754	3	0	THGPLLANFAMV
ORF2	791-802	439.2349	1314.6828	1314.6754	6	0	THGPLLANFAMV
ORF2	807-819	805.4112	1608.8079	1608.808	0	2	RDQRQEEHSVGLR
ORF2	807-819	403.456	1609.7949	1609.7921	2	2	RDQRQEEHSVGLR
ORF2	808-819	727.3609	1452.7072	1452.7069	0	1	DQRQEEHSVGLR
ORF2	808-819	485.2431	1452.7074	1452.7069	0	1	DQRQEEHSVGLR
ORF2	808-819	364.1842	1452.7077	1452.7069	1	1	DQRQEEHSVGLR
ORF2	808-820	537.2772	1608.8097	1608.808	1	2	DQRQEEHSVGLRI
ORF2	811-819	519.2546	1036.4947	1036.4938	1	0	QEEHSVGLR
ORF2	811-819	352.1808	1053.5207	1053.5203	0	0	QEEHSVGLR
ORF2	811-819	352.1809	1053.5209	1053.5203	1	0	QEEHSVGLR
ORF2	811-819	352.181	1053.5213	1053.5203	1	0	QEEHSVGLR
ORF2	811-819	527.7679	1053.5213	1053.5203	1	0	QEEHSVGLR
ORF2	811-819	527.7682	1053.5219	1053.5203	1	0	QEEHSVGLR
ORF2	811-819	352.1813	1053.5222	1053.5203	2	0	QEEHSVGLR
ORF2	811-819	352.1814	1053.5223	1053.5203	2	0	QEEHSVGLR
ORF2	811-819	527.7684	1053.5223	1053.5203	2	0	QEEHSVGLR
ORF2	811-820	398.5389	1192.595	1192.5949	0	1	QEEHSVGLRI
ORF2	811-820	597.3048	1192.5951	1192.5949	0	1	QEEHSVGLRI
ORF2	811-820	398.5391	1192.5955	1192.5949	0	1	QEEHSVGLRI
ORF2	811-820	398.5394	1192.5963	1192.5949	1	1	QEEHSVGLRI
ORF2	811-820	404.214	1209.6203	1209.6214	-1	1	QEEHSVGLRI
ORF2	811-820	605.8181	1209.6217	1209.6214	0	1	QEEHSVGLRI
ORF2	811-820	404.2148	1209.6226	1209.6214	1	1	QEEHSVGLRI
ORF2	811-820	404.215	1209.6233	1209.6214	2	1	QEEHSVGLRI
ORF2	811-820	404.2152	1209.6237	1209.6214	2	1	QEEHSVGLRI
ORF2	811-820	605.8192	1209.6239	1209.6214	2	1	QEEHSVGLRI
ORF2	820-829	366.9043	1097.6912	1097.6921	-1	1	RISTIVGKPI
ORF2	820-829	549.8539	1097.6933	1097.6921	1	1	RISTIVGKPI
ORF2	820-829	366.9054	1097.6944	1097.6921	2	1	RISTIVGKPI
ORF2	821-829	471.8027	941.5909	941.591	0	0	ISTIVGKPI
ORF2	821-829	471.8028	941.591	941.591	0	0	ISTIVGKPI
ORF2	821-829	471.8031	941.5917	941.591	1	0	ISTIVGKPI
ORF2	821-829	471.8033	941.592	941.591	1	0	ISTIVGKPI
ORF2	830-838	512.3006	1022.5867	1022.5834	3	0	ILYMGTVVT
ORF2	830-838	512.3006	1022.5867	1022.5834	3	0	ILYMGTVVT
ORF2	830-838	512.3011	1022.5876	1022.5834	4	0	ILYMGTVVT
ORF2	830-838	512.3013	1022.588	1022.5834	4	0	ILYMGTVVT
ORF2	830-838	520.297	1038.5794	1038.5784	1	0	ILYMGTVVT
ORF2	830-838	520.297	1038.5794	1038.5784	1	0	ILYMGTVVT

ORF2	830-838	520.2974	1038.5802	1038.5784	2	0	ILYMGTVVT
ORF2	830-838	520.2977	1038.5808	1038.5784	2	0	ILYMGTVVT
ORF2	881-907	736.8395	2943.3291	2943.3141	5	0	GPTHRPADAGPTHMHANDFTNTNQMPA
